# Frontiers in quantifying wildlife behavioural responses to chemical pollution

**DOI:** 10.1111/brv.12844

**Published:** 2022-03-01

**Authors:** Michael G. Bertram, Jake M. Martin, Erin S. McCallum, Lesley A. Alton, Jack A. Brand, Bryan W. Brooks, Daniel Cerveny, Jerker Fick, Alex T. Ford, Gustav Hellström, Marcus Michelangeli, Shinichi Nakagawa, Giovanni Polverino, Minna Saaristo, Andrew Sih, Hung Tan, Charles R. Tyler, Bob B.M. Wong, Tomas Brodin

**Affiliations:** ^1^ Department of Wildlife, Fish, and Environmental Studies Swedish University of Agricultural Sciences Skogsmarksgränd 17 Umeå Västerbotten SE‐907 36 Sweden; ^2^ School of Biological Sciences Monash University 25 Rainforest Walk Melbourne Victoria 3800 Australia; ^3^ Department of Environmental Science Baylor University One Bear Place Waco Texas 76798‐7266 U.S.A.; ^4^ Faculty of Fisheries and Protection of Waters, South Bohemian Research Center of Aquaculture and Biodiversity of Hydrocenoses University of South Bohemia in Ceske Budejovice Zátiší 728/II Vodnany 389 25 Czech Republic; ^5^ Department of Chemistry Umeå University Linnaeus väg 10 Umeå Västerbotten SE‐907 36 Sweden; ^6^ Institute of Marine Sciences University of Portsmouth Winston Churchill Avenue, Portsmouth Hampshire PO1 2UP U.K.; ^7^ Department of Environmental Science and Policy University of California 350 E Quad, Davis California CA 95616 U.S.A.; ^8^ Evolution & Ecology Research Centre, School of Biological, Earth and Environmental Sciences University of New South Wales, Biological Sciences West (D26) Sydney NSW 2052 Australia; ^9^ Centre for Evolutionary Biology, School of Biological Sciences University of Western Australia 35 Stirling Highway Perth WA 6009 Australia; ^10^ Department of Ecological and Biological Sciences Tuscia University Via S.M. in Gradi n.4 Viterbo Lazio 01100 Italy; ^11^ Environment Protection Authority Victoria EPA Science 2 Terrace Way Macleod Victoria 3085 Australia; ^12^ Biosciences, College of Life and Environmental Sciences University of Exeter Stocker Road Exeter Devon EX4 4QD U.K.

**Keywords:** animal, behaviour, contaminant, ecology, ecotoxicology, environmental change, fitness, pollutant, population, wildlife

## Abstract

Animal behaviour is remarkably sensitive to disruption by chemical pollution, with widespread implications for ecological and evolutionary processes in contaminated wildlife populations. However, conventional approaches applied to study the impacts of chemical pollutants on wildlife behaviour seldom address the complexity of natural environments in which contamination occurs. The aim of this review is to guide the rapidly developing field of behavioural ecotoxicology towards increased environmental realism, ecological complexity, and mechanistic understanding. We identify research areas in ecology that to date have been largely overlooked within behavioural ecotoxicology but which promise to yield valuable insights, including within‐ and among‐individual variation, social networks and collective behaviour, and multi‐stressor interactions. Further, we feature methodological and technological innovations that enable the collection of data on pollutant‐induced behavioural changes at an unprecedented resolution and scale in the laboratory and the field. In an era of rapid environmental change, there is an urgent need to advance our understanding of the real‐world impacts of chemical pollution on wildlife behaviour. This review therefore provides a roadmap of the major outstanding questions in behavioural ecotoxicology and highlights the need for increased cross‐talk with other disciplines in order to find the answers.

## I INTRODUCTION

Chemical pollution represents a leading threat to human health, wildlife, and ecosystems around the world (Landrigan *et al*., 2018). In fact, increases in the diversity and volume of novel synthetic chemicals released into the environment now far outpace other key drivers of global change (e.g. rising atmospheric CO_2_, habitat loss; Bernhardt, Rosi & Gessner, 2017). This trend is the result of rapidly increasing chemical use, with the global chemicals industry, currently valued at >US$5 trillion, projected to double in size between 2017 and 2030 (UNEP, 2019). The spread of novel environmental contaminants is concerning given that many ecosystems are already inundated with myriad organic and inorganic chemicals released by human activities, including metals, pesticides, pharmaceuticals, and per‐ and polyfluoroalkyl substances (PFAS) (Bertram *et al*., 2022). Moreover, chemical pollution continues to accelerate large‐scale wildlife losses (Groh *et al*., 2022), including dramatic declines in the abundance and biodiversity of insects (Sánchez‐Bayo & Wyckhuys, 2019), avifauna (Rosenberg *et al*., 2019), and aquatic species (Tickner *et al*., 2020).

Adverse impacts of chemical pollution on wildlife are not limited to mortality at acutely toxic levels. Indeed, exposure to environmental pollutants at sublethal concentrations acts as an often‐cryptic driver of wildlife declines by disrupting a wide range of fundamental biological processes [e.g. development (Gore *et al*., 2015); reproduction (Aulsebrook *et al*., 2020)]. In this regard, a growing body of research has demonstrated that chemical pollutants can have both direct and indirect impacts at multiple levels of organisation by altering animal behaviour (reviewed in Saaristo *et al*., 2018). In fact, behaviour can be remarkably sensitive to perturbation by even low pollutant concentrations, and is often disturbed at much lower exposure levels than more conventional endpoints in ecotoxicology, such as development, reproduction, and mortality, which is routinely estimated using LC_50_ (i.e. the concentration at which 50% of the animals in an exposed population are expected to die) (Melvin & Wilson, 2013). This is alarming given that behaviour represents the link between an organism and its environment, meaning that an inability to appropriately produce and maintain behaviour can have dire consequences for individual‐ and population‐level fitness (Wong & Candolin, 2015).

Clearly, there is an urgent need to better establish the causes and consequences of pollutant‐induced behavioural changes in wildlife (discussed in Peterson *et al*., 2017). However, conventional approaches applied in behavioural ecotoxicology are often insufficient to address the complexity of real‐world exposure scenarios (Pyle & Ford, 2017), that is exposing individuals of a single species to a single contaminant and measuring movement in a confined arena. This limits our ability to achieve meaningful biological interpretations of pollutant impacts at the individual level. Moreover, such approaches hamper the accurate extrapolation of findings to predict effects on populations and communities, which is vital to environmental protection efforts (Ford *et al*., 2021).

At the same time, significant methodological and technological innovations promise to transform the study of wildlife behavioural responses to pollutants, originating from within behavioural ecotoxicology and related disciplines such as analytical, environmental, and computational chemistry, comparative toxicology, behavioural ecology, collective behaviour, movement ecology, automated sensing, and computer vision. These advances open the door to the collection of data of high environmental and ecological relevance at an unprecedented resolution and scale in both the laboratory and the field. Crucially, this is enabling behavioural ecotoxicologists to confront the formidable complexity that often characterises real‐world exposure scenarios (e.g. chemical interactions, chemical and non‐chemical stressor interactions, complex and/or large‐scale behaviours). Indeed, recent work embracing these innovations has revealed effects of pollutants on behavioural processes that were previously not possible to capture using conventional approaches. These range from neurotoxic insecticide‐induced migration delays in songbirds (Eng, Stutchbury & Morrissey, 2019) to the breakdown of behavioural diversity in fish populations exposed to pharmaceutical pollution (Polverino *et al*., 2021).

The aim of this review is to guide the field of behavioural ecotoxicology towards increased environmental realism, ecological complexity, and mechanistic understanding by highlighting major research opportunities and experimental innovations. First, we examine how recent advances in analytical and environmental chemistry can increase environmental realism and experimental quality in behavioural ecotoxicology, and discuss underused approaches to prioritising contaminants of concern for behavioural testing. We then argue for targeting behavioural endpoints of high ecological significance, and outline concepts in ecology that have been broadly overlooked in behavioural ecotoxicology to date but which have the potential to provide key insights. We go on to present a suite of cutting‐edge tools and technologies that can be incorporated into behavioural ecotoxicology studies during design, implementation, and analysis. Further, we consider developments in statistical sophistication necessary to evaluate behavioural impacts of pollutants occurring in complex systems. Finally, we provide open‐access and freely available tools and resources, wherever possible, in order to increase the accessibility of behavioural ecotoxicology research.

## II INCREASING ENVIRONMENTAL REALISM

Here, we outline how recent advances in analytical and environmental chemistry can be harnessed to enhance environmental realism and experimental quality in behavioural ecotoxicology, and discuss underused approaches for prioritising contaminants and mixtures of concern for behavioural testing.

### (1) Confronting real‐world exposure scenarios

Given the sheer number of chemicals in commerce, one of the challenges we face is how to identify what potentially behaviour‐modifying chemicals and mixtures are present in the environment and at what levels. There are at least 350,000 chemicals and chemical combinations registered for use around the world (Wang *et al*., 2020), several thousand of which have been detected in the environment (Hollender *et al*., 2017). However, regulatory monitoring is still limited to a small selection of well‐known contaminants, representing only a fraction of overall chemical and mixture risk (discussed in Brack *et al*., 2019a). For example, the United States Clean Water Act regulates 126 priority pollutants (EPA, 2021a), while the European Water Framework Directive is based on the analysis of 45 priority substances, as well as pollutants (~300 total) defined nationally by the different European Union member states (European Union, 2013). This limitation is partly a consequence of traditional environmental surveillance methodologies relying on targeted analyses of contaminants (discussed in Brack *et al*., 2019b), which requires that compounds are already ‘known’ and that chemical analytical standards are available to support their measurement. Fortunately, rapid advances in analytical and environmental chemistry, such as non‐targeted screening using high‐resolution mass spectrometry coupled to chromatography, now allow for near‐simultaneous identification of thousands of contaminants that are not preselected for study across a range of environmental compartments (e.g. water, air, dust, soil) and biological matrices (e.g. tissue, blood, plasma) (Hollender *et al*., 2017; McCord, Groff II & Sobus, 2022). The sensitivity of analytical instrumentation for both targeted and non‐targeted analyses has also increased markedly, allowing for the detection of even trace concentrations (e.g. pg/l or kg, ng/l or kg) of chemicals that could cause sub‐lethal changes to animal behaviour (Jiang & Li, 2020). However, equipment and analysis costs are still a limiting factor in the widespread application of these approaches (see Fernandez, André & Cardeal, 2020). Thankfully, data on complex exposure scenarios in the wild are also increasingly accessible due to the availability of large‐scale, open‐access databases documenting the occurrence of environmental contaminants, for example the EPA NCOD Database (EPA, 2021b), the NORMAN EMPODAT Database (NORMAN, 2022), the UBA Pharmaceuticals in the Environment Database (UBA, 2021), and the Global Monitoring of Pharmaceuticals Project (Wilkinson *et al*. 2022). Taken together, these developments increasingly allow behavioural ecotoxicologists to place their work in a real‐world context when targeting contaminants and mixtures of concern (e.g. based on environmental prevalence, persistence, co‐occurrence, and/or relevance to particular species or research questions). This targeted approach is especially important given that testing all relevant chemicals and combinations of chemicals for potential behavioural effects would be prohibitively costly, inefficient, and ethically problematic. Moreover, these advances facilitate extreme precision, accuracy, and breadth in characterising and validating exposure scenarios (e.g. confirming contaminants and transformation products in exposure media and/or experimental animal tissues).

### (2) Prioritising contaminants of concern

The next major challenge is identifying and prioritising contaminants and mixtures that are likely to have deleterious effects on wildlife behaviour. To this end, we must look beyond traditional laboratory exposure experiments alone, again adopting a targeted approach given that more chemicals exist than can be adequately examined in a timely manner. It is therefore crucial to *predict* how chemicals interact with biological systems, which requires developing predictive approaches to identify and prioritise contaminants and mixtures that are likely (or not) to alter behaviour. In addition to facilitating the analytical chemistry advances discussed previously, computational chemistry can help to identify inherent chemical attributes and properties that are likely to interact with specific biomolecules and elicit downstream biological and behavioural responses in organisms. For example, an explosion of molecular genetic information across phyla is facilitating *in silico* advances in predictive toxicology and pharmacology during drug development (Raies & Bajic, 2016). Such approaches are translatable from human to environmental protection efforts when the structural alerts for specific chemicals align with predictions of evolutionarily conserved molecular initiating events (MIEs, i.e. a molecular interaction between a chemical and a specific biomolecule; LaLone *et al*., 2016). These MIEs may then lead to a sequential series of higher‐order effects (e.g. gene activation, changed cell signalling, disturbed homeostasis, altered tissue development and/or function) across biological scales (e.g. molecular, cellular, tissue, and/or organ levels). This may produce adverse outcomes – for instance, on behaviour – of relevance to individuals and populations [summarised in the ‘Adverse Outcome Pathway’ (AOP) concept; Ankley *et al*., 2010]. Adding a layer of complexity, AOPs can also be used to predict mixture effects (reviewed in Escher *et al*., 2017), which may be produced by combinations of chemicals that act *via* the same or different MIEs, and could include additive, antagonistic, or synergistic interactive effects on animal behaviour. Supporting this approach, large‐scale computational toxicology initiatives are examining thousands of chemicals with hundreds of targets in *in vitro* models to identify MIEs that may cause adverse outcomes in the environment (e.g. Tox21, 2020; ToxCast, 2020; ToxPi, 2021). Although very little integration of computational chemistry, comparative toxicology, and behavioural ecotoxicology has occurred to date, this approach could be extremely valuable in facilitating effective prioritisation of contaminants of concern for behavioural testing, and promises to improve mechanistic understanding. Moreover, because groups of chemicals within contaminant classes often act on the same or similar physiological pathways and molecular mechanisms (e.g. serotonin re‐uptake inhibiting pharmaceuticals; Gunnarsson *et al*., 2019), this targeted approach promises to reduce substantially the number of candidate chemicals and mixtures for behavioural testing.

## III ADDRESSING ECOLOGICAL COMPLEXITY

A common critique of behavioural ecotoxicology research is the perception that such studies lack sufficient ecological relevance to be applied to real‐world scenarios (discussed in Ågerstrand *et al*., 2020). This is true even though, when assessed correctly, behavioural responses can be invaluable in predicting higher‐order population‐ and community‐level outcomes (Saaristo *et al*., 2018). Here, we outline practices to improve ecological realism in behavioural ecotoxicology research and highlight seldom‐studied but significant new research avenues in this field.

### (1) Key experimental design considerations

When designing studies testing the potential behavioural impacts of contaminants, how do we decide which behaviours we should test and how to test them most appropriately? Most importantly, behaviours need to be ecologically relevant to the focal species and must be biologically targeted by the contaminant(s) in question *via* some direct or indirect mechanism(s) of action, and/or predicted to impact an organism's spatial pollution attraction/avoidance (discussed in Araújo *et al*., 2020). Contaminants that are expected to affect fundamental behaviours for a species' ecology and fitness should be prioritised, as these will likely have more immediate and adverse impacts. For instance, contaminants that target social‐cue mechanisms may disproportionately affect organisms that rely on social information and/or the ability to form cohesive groups (e.g. schools, flocks) that are essential to their antipredator behaviour, feeding and foraging, and mating biology (e.g. Ward *et al*., 2008; Martin *et al*., 2019; Mason *et al*., 2021). Similarly, certain pollutants might interfere with a species' ability to avoid predators by targeting neural pathways involved in predator recognition (e.g. Polo‐Cavia, Burraco & Gomez‐Mestre, 2016) or that support locomotion for escape (e.g. Sievers *et al*., 2018). Further, behavioural experiments should ideally be tailored to mimic the species' natural social environment and habitat (or be conducted in the wild; see Section IV.2). Social species, for instance, should be tested in the presence of conspecifics (see Martin & McCallum, 2021), both to increase ecological relevance and because of the known impacts of social isolation on animal physiology and behaviour (e.g. Shams, Chatterjee & Gerlai, 2015; Tunbak *et al*., 2020; Munson, Michelangeli & Sih, 2021). Likewise, species that rely on specific environmental resources as part of their behavioural repertoire should be tested with those resources (e.g. refuge structures, substrate, perches), and if movement‐related behaviours are being measured then adequately sized experimental environments should be provided for natural space use and avoidance behaviours to occur. These details will vary from species to species but their absence may lead to unnatural behaviours and erroneous biological interpretations.

### (2) Novel research directions in behavioural ecotoxicology: what are the knowns and the unknowns?

**Fig. 1:**
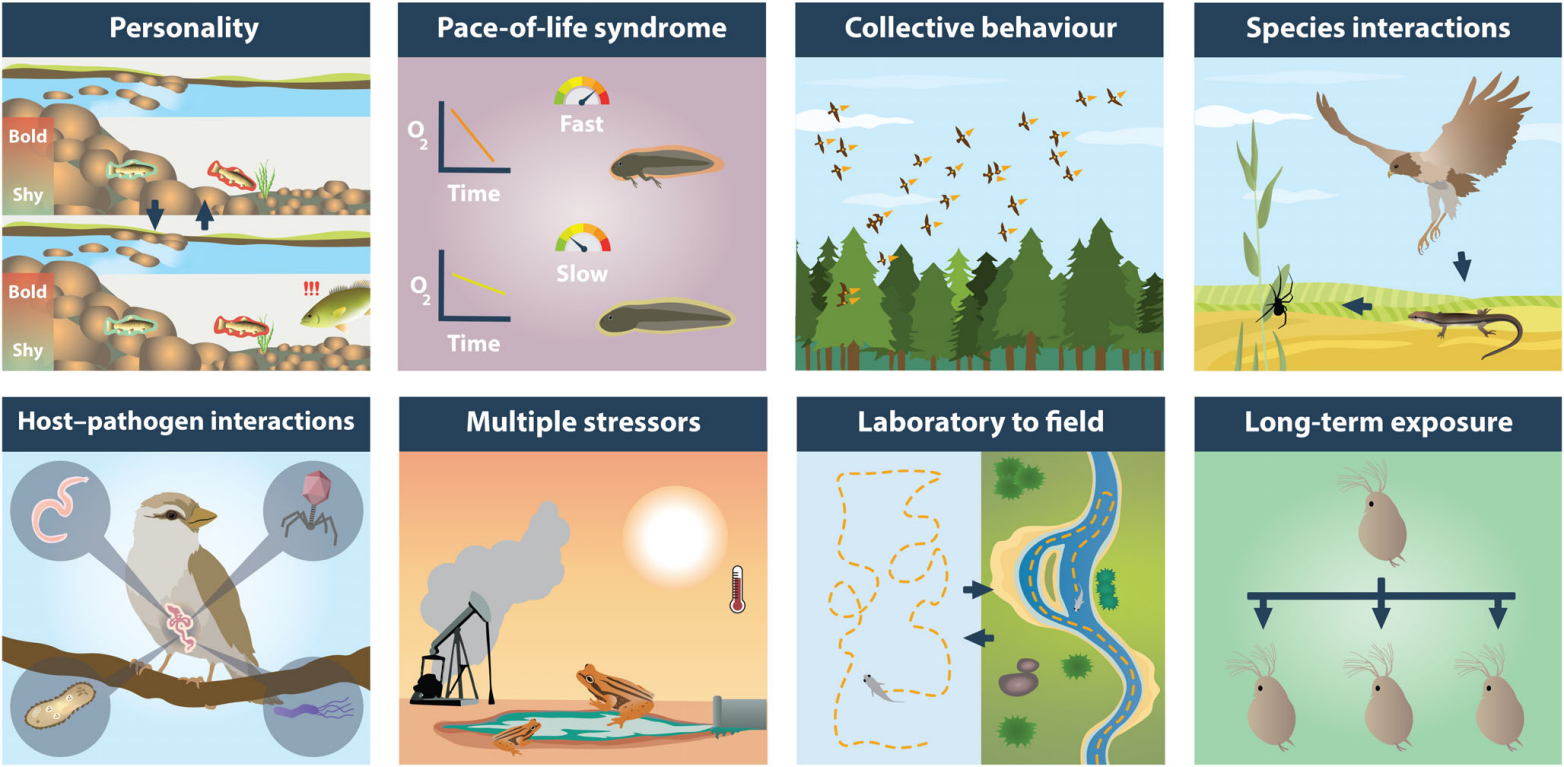
Important future avenues for research investigating behavioural impacts of chemical pollution on wildlife, which have received relatively little attention in behavioural ecotoxicology, especially when compared with more conventional ecotoxicological endpoints (e.g. development, reproduction, and LC_50_). Examples of studies that have been conducted in each of these research areas to date are provided in Table 1.

Keeping the above experimental considerations in mind, we next feature a range of future research avenues that, despite being of high ecological significance, have received relatively little attention in behavioural ecotoxicology (Fig. 1; see also Table 1 for examples of studies that have been carried out in each of these research areas to date). We by no means see this as an exhaustive list or as mutually exclusive groupings. In fact, integrating across these groupings will only help to address the complexities of how pollutant exposure affects animals in the wild.

**Table 1:** Research in behavioural ecotoxicology has increased markedly in ecological realism, especially in recent years. This includes studies conducted within each of the key areas for future research identified in Fig. 1, although such studies are still relatively rare. Examples of these studies are provided here, grouped by research area

Research area	Compound(s)/stressor(s)	Species	Major result(s)	Reference
**Personality** Behavioural differences between individuals that are consistent over time and across contexts (see Montiglio & Royauté, 2014; Jacquin *et al*., 2020)	Phosmet (organophosphate insecticide)	Jumping spider (*Eris militaris*)	Repeatability of personality traits declined in the exposed group, mostly mediated by an increase in within‐individual variance.	Royauté, Buddle & Vincent (2015)
	Esfenvalerate (pyrethroid insecticide)	Damselfly (*Coenagrion puella*)	Exposure changed average activity and behavioural covariation (activity and boldness) but not behavioural repeatability.	Tüzün *et al*. (2017)
	Mixture of metals, mainly lead, copper, and zinc	Great tit (*Parus major*)	Exploration behaviour and aggressiveness during nest defence were repeatable across years. Birds with high levels of lead in their blood and high levels of multiple metals in their feathers exhibited slower exploration behaviour but no effect of exposure was seen on aggressiveness.	Grunst *et al*. (2019)
	Fluoxetine (antidepressant pharmaceutical)	Guppy (*Poecilia reticulata*)	Homogenised individuals' activity (i.e. reduced consistent variation between individuals).	Tan *et al*. (2020)
	Fluoxetine (antidepressant pharmaceutical)	Guppy (*Poecilia reticulata*)	Homogenised individuals' activity (i.e. reduced variation between but not within individuals).	Polverino *et al*. (2021)
**Pace‐of‐life syndrome** The correlation between an individual's average behaviour (e.g. activity) and averages of its other phenotypic traits (e.g. metabolic rate, growth rate), with all traits significantly repeatable after repeated measures.	Chlorpyrifos (organophosphate insecticide)	*Ischnura* damselfly species (*I. elegans*, *I. genei*, *I. graellsii* and *I. pumilio*)	Exposure affected covariation of life‐history and boldness in the most fast‐lived species (*I. pumilio*).	Debecker *et al*. (2016)
	Zinc (heavy metal)	Blue‐tailed damselfly (*Ischnura elegans*)	Fast pace‐of‐life was associated with higher zinc sensitivity. Zinc exposure made larvae less active, less exploratory, and less risk‐taking. Exposure to zinc did not change the covariation patterns between traits (behavioural and physiological).	Debecker & Stoks (2019)
	Fluoxetine (antidepressant pharmaceutical)	Fairy shrimp (*Branchipodopsis wolf*)	Fluoxetine disrupted sex‐specific relationships between body size (proxy for growth rate) and activity.	Thoré, Brendonck & Pinceel (2021b)
**Collective behaviour** A form of social behaviour involving the coordinated behaviour of groups of similar individuals, and the emergent properties of these groups.	4‐nonylphenol (endocrine‐disrupting chemical)	Banded killifish (*Fundulus diaphanus*)	Unexposed fish oriented away from dosed conspecifics. Shoals of all exposed fish had larger nearest‐neighbour distances (less‐tight shoals).	Ward *et al*. (2008)
	Imidacloprid (neonicotinoid insecticide)	Bumblebee (*Bombus impatiens*)	Impaired nursing behaviour and altered social and spatial dynamics of workers within nests.	Crall *et al*. (2018)
	Crude oil	Atlantic croaker (*Micropogonias undulatus*)	Reduced shoal cohesion in shoals with all exposed fish and in shoals with only one exposed fish.	Armstrong *et al*. (2019)
	Benzo[*a*]pyrene (polycyclic aromatic hydrocarbon; PAH)	Zebrafish (*Danio rerio*)	Increased inter‐individual distances in exposed shoals. Exposed shoals moved less overall.	Hamilton *et al*. (2021)
	Oxazepam (anxiolytic pharmaceutical)	Brown trout (*Salmo trutta*)	Fish were less aggressive at higher doses and subordinate fish became more competitively successful at low doses (dominant and subordinate fish affected differently).	McCallum *et al*. (2021)
**Species interactions** Behavioural interactions between individuals of different species, such as predator–prey and competitive interactions (see Saaristo *et al*., 2018; Fleeger, 2020; Fisher *et al*., 2021)	Carbaryl (carbamate insecticide), malathion (organophosphate insecticide)	Amphibian prey (gray treefrog, *Hyla versicolor*; green frog, *Rana clamitans*; American bullfrog, *R. catesbeiana*) and a predator (red‐spotted newt, *Notophthalmus viridescens*)	Exposure to either insecticide reduced the activity of all three tadpole prey species, and reduced the predation rate of newts on one tadpole species.	Relyea & Edwards (2010)
	Urban and industrial contamination, including polychlorinated biphenyls (PCBs), polycyclic aromatic hydrocarbons (PAHs), dioxins, and metals (e.g. copper, lead, zinc)	Killifish (*Fundulus heteroclitus*), bluefish (*Pomatomus saltatrix*), fiddler crab (*Uca pugnax*), blue crab (*Callinectes sapidus*), grass shrimp (*Palaemonetes pugio*)	At contaminated sites, all five species showed reduced activity and feeding. Complex behavioural changes were also seen within species due to contamination, including reduced predator avoidance in killifish but increased predator avoidance in fiddler crabs and blue crabs.	Weis *et al*. (2011)
	Trifloxystrobin (TFS, strobilurin fungicide)	Eel (*Synbranchus marmoratus*), four anuran prey species (*Rhinella arenarum*, *Physalaemus santafecinus*, *Leptodactylus latrans*, *Elachistocleis bicolor*)	Exposure altered the outcome of eel–tadpole interactions by decreasing prey movement and prey detection, increasing tadpole survival. Eels preyed selectively upon non‐exposed tadpoles.	Junges *et al*. (2012)
	17β‐oestradiol (E2, oestrogen steroid hormone)	Fathead minnow (*Pimephales promelas*), bluegill sunfish (*Lepomis macrochirus*)	Exposure reduced anti‐predator escape behaviour of larval minnows and they were more likely to be predated by a sunfish predator.	Rearick *et al*. (2018)
	Dichlorodiphenyltrichloroethane (DDT, organochlorine insecticide)	African clawed frog (*Xenopus laevis*), mosquito (*Culex* sp.)	Significant exposure × prey cue interaction. Exposure reduced frog foraging behaviour towards live prey cues, although no effect was seen in response to olfactory prey cues. Mosquito larvae exhibited reduced antipredator behaviour.	South *et al*. (2019)
**Host–pathogen interactions** The interactions between microbes (e.g. parasites, bacteria, viruses) and their host organism(s), which may alter the behaviour of the host.	Imidacloprid (neonicotinoid insecticide), entomopathogenic fungi (*Metarhizium robertsii* and *Beauveria bassiana*)	Citrus root weevil (*Diaprepes abbreviatus*)	Application of either fungus had no effect on the movement of larvae in soil, although insecticide exposure was found to impair larval movement. Moreover, exposure to both imidacloprid and a fungus acted synergistically to produce more severe impairment to larval movement.	Quintela & McCoy (1998)
	Chlorpyrifos (organophosphate insecticide), trematode parasite (*Euhaplorchis californiensis*)	California killifish (*Fundulus parvipinnis*)	Insecticide exposure reduced activity and decreased average swimming speed following a simulated predator attack. No singular or interactive effects of parasite infection were observed.	Renick *et al*. (2016)
	Mixture of metals (cadmium, copper, and zinc), immune challenge (antigen mixture mimicking a parasite infection)	Gudgeon (*Gobio occitaniae*)	Single stressors increased immune defences and oxidative stress at the expense of body mass (metal contamination) or swimming activity (immune challenge). Multiple stressors produced fewer interactive effects than expected but primarily resulted in antagonistic effects on swimming activity.	Petitjean *et al*. (2021)
**Multiple stressors** The potentially interacting effects of combined exposure to multiple contaminants (within and/or across contaminant classes), or to contaminants and other external stressors (e.g. temperature, light, noise) (see Halfwerk & Slabbekoorn, 2015; Hale, Piggott & Swearer, 2017; Jacquin *et al*., 2020)	Copper (heavy metal), imidacloprid (insecticide)	Spotted marsh frog (*Limnodynastes tasmaniensis*)	Copper increased erratic swimming at the lower imidacloprid concentration. Limited overall evidence for interactive effects (both stressors produced independent effects).	Sievers *et al*. (2018)
	Chlorpyrifos (organophosphate insecticide), flow speed	California killifish (*Fundulus parvipinnis*), polychaete worm (*Polydora cornuta*)	Contamination and differing flow speeds resulted in complex effects on predator–prey interactions, including reducing prey‐patch selection in contaminated killifish and reducing feeding behaviour in worms.	Hayman *et al*. (2019)
	17β‐trenbolone (androgenic steroid), temperature	Eastern mosquitofish (*Gambusia holbrooki*)	Contamination increased boldness, with effects of exposure on some behaviours (i.e. exploration and predator‐escape behaviour) being dependent on temperature.	Lagesson *et al*. (2019)
	Fluoxetine (antidepressant pharmaceutical), acute temperature stress	Guppy (*Poecilia reticulata*)	No evidence for interactive effects on reproductive behaviours and activity levels (both stressors produced independent effects).	Wiles *et al*. (2020)
	Chlorpyrifos (organophosphate insecticide), acute, developmental, and transgenerational warming	Mosquito (*Culex pipiens*)	Particularly developmental and transgenerational warming reduced larvae antipredator behaviours. Contamination decreased heat tolerance and antipredator behaviours.	Meng *et al*. (2021)
**Laboratory to field** Using paired laboratory‐ and field‐based approaches to investigate potentially behaviour‐modifying effects of contaminants. *Note*: also included here are semi‐field and field‐based studies without a paired laboratory component (see Hellström *et al*., 2016b; Saaristo *et al*., 2018)	Urban and industrial contamination, including copper, cadmium, and polycyclic aromatic hydrocarbons (PAHs).	Round goby (*Neogobius melanostomus*)	In the laboratory, fish collected from contaminated sites exhibited reduced activity and exploration, although this was not reflected in distance moved in a mark–recapture field study.	Marentette *et al*. (2012)
	Neonicotinoid insecticide mixture (thiamethoxam and imidacloprid)	European honey bee (*Apis mellifera*)	Individual honeybees near treated fields disappeared more quickly but this was buffered by the colonies' demographic regulation response.	Henry *et al*. (2015)
	Oxazepam (anxiolytic pharmaceutical)	Atlantic salmon (*Salmo salar*)	Promoted downward migratory behaviour in the laboratory and in a natural river tributary.	Hellström *et al*. (2016a)
	Oxazepam (anxiolytic pharmaceutical)	European perch (*Perca fluviatilis*)	Increased boldness and activity both in the laboratory and in a lake ecosystem.	Klaminder *et al*. (2016)
	Clothianidin (neonicotinoid insecticide)	Bumblebee (*Bombus terrestris audax*)	In a semi‐field experiment, exposure produced subtle changes in patterns of foraging activity and pollen foraging, with a colony census at the end of the experiment revealing that treated colonies had fewer adults (workers, drones, and gynes) compared to control colonies.	Arce *et al*. (2017)
**Long‐term exposure** Examining potential behavioural effects of long‐term exposure to contaminants, including chronic, transgenerational, and multigenerational exposures.	Carbamazepine (anticonvulsant pharmaceutical), gemfibrozil (blood lipid‐regulating pharmaceutical)	Zebrafish (*Danio rerio*)	Parental exposure to either drug reduced male courtship behaviour in unexposed offspring. Effects on courtship displays were compound‐specific.	Galus *et al*. (2014)
	Fluoxetine (antidepressant pharmaceutical)	Zebrafish (*Danio rerio*)	Developmental exposure produced hypocortisolism and reduced exploratory behaviours in two consecutive generations of unexposed descendants.	Vera‐Chang *et al*. (2018)
	Lambda‐cyhalothrin (LCT, pyrethroid insecticide)	Mustard leaf beetle (*Phaedon cochleariae*)	Parental exposure altered aspects of mating behaviour in both the parental generation and unexposed offspring.	Müller, Römer & Müller (2019)
	Urban contaminant mixture, including bisphenol‐A (BPA, xenoestrogen), N,N‐diethyl‐meta‐toluamide (DEET, insect repellent), and 4‐nonylphenol (xenoestrogen)	Fathead minnow (*Pimephales promelas*)	Exposure for three generations altered the behaviour (foraging, courtship, boldness) of larvae and adults, which were magnified in the F_1_ and F_2_ generations.	Swank *et al*. (2021)
	Fluoxetine (antidepressant pharmaceutical) and 3,4‐dichloroaniline (pesticide)	Turquoise killifish (*Nothobranchius furzeri*)	Behavioural effects differed in single‐chemical *versus* mixture exposure treatments, and across two successive generations.	Thoré *et al*. (2021)

At the individual level, behavioural ecotoxicology research to date has largely focused on how exposure to chemicals alters the average level of ecologically relevant behaviours (e.g. activity, aggression, boldness). Such observations have been pragmatic from a laboratory experimental design perspective but often ignore behavioural variation. This is concerning because phenotypic plasticity, that is an individual's capacity for phenotypic variation under different environments, is a key determinant of performance under fluctuating conditions within their lifetime (often called ‘acclimation’; see Donelson *et al*., 2019). Moreover, phenotypic variation among individuals is fundamental for adaptation (and evolution) to occur, meaning that the extent of variation within a population is an important indicator of how buffered that population will be to environmental change, including chemical pollution (Sih, Ferrari & Harris, 2011). Accordingly, the field of behavioural ecology has seen a shift towards quantifying and understanding consistent within‐ and among‐individual behavioural variation (i.e. animal personality; Sih, Bell & Johnson, 2004). This includes both phenotypic plasticity and other sources of behavioural variation such as circadian/circannual rhythms (e.g. Melvin, 2017; Thoré, Brendonck & Pinceel, 2021a), and life‐history events (e.g. Thoré *et al*., 2019). This has produced new insights into how consistent individual behavioural differences are correlated with key traits related to behaviour, such as cognitive ability (Sih & Del Giudice, 2012; Griffin, Guillette & Healy, 2015), metabolic rate (Careau *et al*., 2008; Biro & Stamps, 2010), and space‐use patterns (Spiegel *et al*., 2017; Michelangeli *et al*., 2021). Furthermore, individual behavioural variation is heritable (Dochtermann, Schwab & Sih, 2015) but can also be shaped by experience *via* transgenerational and developmental phenotypic plasticity (Groothuis & Taborsky, 2015), including exposure to chemical pollutants (Tüzün *et al*., 2017). Importantly, differences in behaviour among individuals may also lead to variation in sensitivity and exposure to chemical pollutants. For example, recent research has demonstrated that social status modulates the extent of uptake of the pharmaceutical pollutant oxazepam by juvenile brown trout (*Salmo trutta*), with subordinate fish absorbing more of the drug than dominant ones, likely due to higher ventilation and respiration rates (McCallum *et al*., 2021). Given that only a relatively small number of recent studies have examined how chemical stressors alter aspects of behavioural variation (e.g. Tüzün *et al*., 2017; Polverino *et al*., 2021), or how consistent individual differences affect behavioural responses to chemical stressors (Nanninga, Scott & Manica, 2020), the integration of behavioural ecotoxicology and consistent individual variation is clearly an important direction for future study (see Montiglio & Royauté, 2014).

Scaling up to the group and population levels, we know that animals display a range of social phenotypes (Gartland *et al*., 2021) and that interactions among conspecifics can underlie many aspects of animal fitness (e.g. territory defence, reproduction, parental care). Yet very little attention has been given to understanding how contaminants might affect animal groups and intraspecific interactions (beyond simple dyads). As such, another significant future direction will be to measure the impact of chemical pollutants in natural social settings on endpoints like: (*i*) social network structure, including the formation and consequences of dominance hierarchies; (*ii*) collective behaviours such as group shoaling, flocking, foraging, or collective decision‐making; (*iii*) how individual traits (e.g. personality variation) affect group or collective behaviour; (*iv*) animal contests over valued resources (e.g. territory, mates); and (*v*) classic reproductive behaviours like courtship, mating and alternative strategies, and parental care. This work can also operate beyond a single generation because an individual's behaviour can be shaped by both past and current exposure, as well as past conspecific experiences *via* indirect maternal/paternal effects (Bell & Hellmann, 2019; Donelan *et al*., 2020).

We also need to look beyond any single species to answer questions concerning multi‐species interactions and how behaviour is affected by other ecological processes across environmental gradients. These types of questions will allow us to better approximate the complexities of natural environments and might include, for example, uncovering whether and how contaminants could decouple or change host–parasite or host–pathogen relationships (Blanken, van Langevelde & van Dooremalen, 2015; Rumschlag *et al*., 2019), alter interspecific information transfer or eavesdropping (Gil *et al*., 2018), and affect predator–prey interactions (Weis & Candelmo, 2012; Hayden *et al*., 2015). Moreover, considering that various pollutants (e.g. psychoactive pharmaceuticals) have been shown to alter predator avoidance and escape behaviours in prey species (e.g. Polo‐Cavia, Burraco & Gomez‐Mestre, 2016; Martin *et al*., 2017), key questions that have received surprisingly little attention to date are whether exposure of predators and/or prey to (the same or different) contaminants might shift the ‘landscape of fear’ (Gaynor *et al*., 2019), or shape animal communities and trophic cascades through altered patterns of competition and/or consumption (e.g. Rohr, Kerby & Sih, 2006; Weis *et al*., 2011).

Finally, to reveal patterns of effects of chemicals on individuals, animal groups, and whole community/ecosystem outcomes, a crucial need is to understand the relative impact of chemical stressors and other abiotic and biotic stressors that can have unexpected and complex effects on animal behaviour, that is we need a multi‐stressor approach (Orr *et al*., 2020). Numerous studies show that even very low levels of chemical stressors can severely reduce fitness if combined with low food levels (e.g. Rohr *et al*., 2004), predation risk (Relyea, 2003), high parasite load (e.g. Rumschlag *et al*., 2019), or variable temperature (e.g. Brown *et al*., 2015; Delnat *et al*., 2019). Yet relatively few studies have examined how behaviour might mediate these multi‐stressor outcomes *via* animal dispersal or avoidance, seasonal or diurnal shifts in behaviour, or increasing/decreasing behaviours to compensate for changing metabolic demands (e.g. a foraging–detoxification trade‐off). Among species, some of the variation in negative impacts of chemical stressors will be due to differences in physiology that underlie inherent differences in vulnerability to exposure. However, differences in behavioural responses will likely play a major role in explaining dissimilar exposure to chemicals, and in the ability of animals to compensate behaviourally, for example by increasing energy intake or *via* social buffering. Thus, more accurate behavioural assessments of chemical pollutants will often require a multi‐stressor approach in order to assess the simultaneous or sequential effects of chemicals with other key environmental factors within ecologically relevant conditions.

## IV TOOLS AND TECHNOLOGIES FOR ADVANCING BEHAVIOURAL ECOTOXICOLOGY

**Fig. 2:**
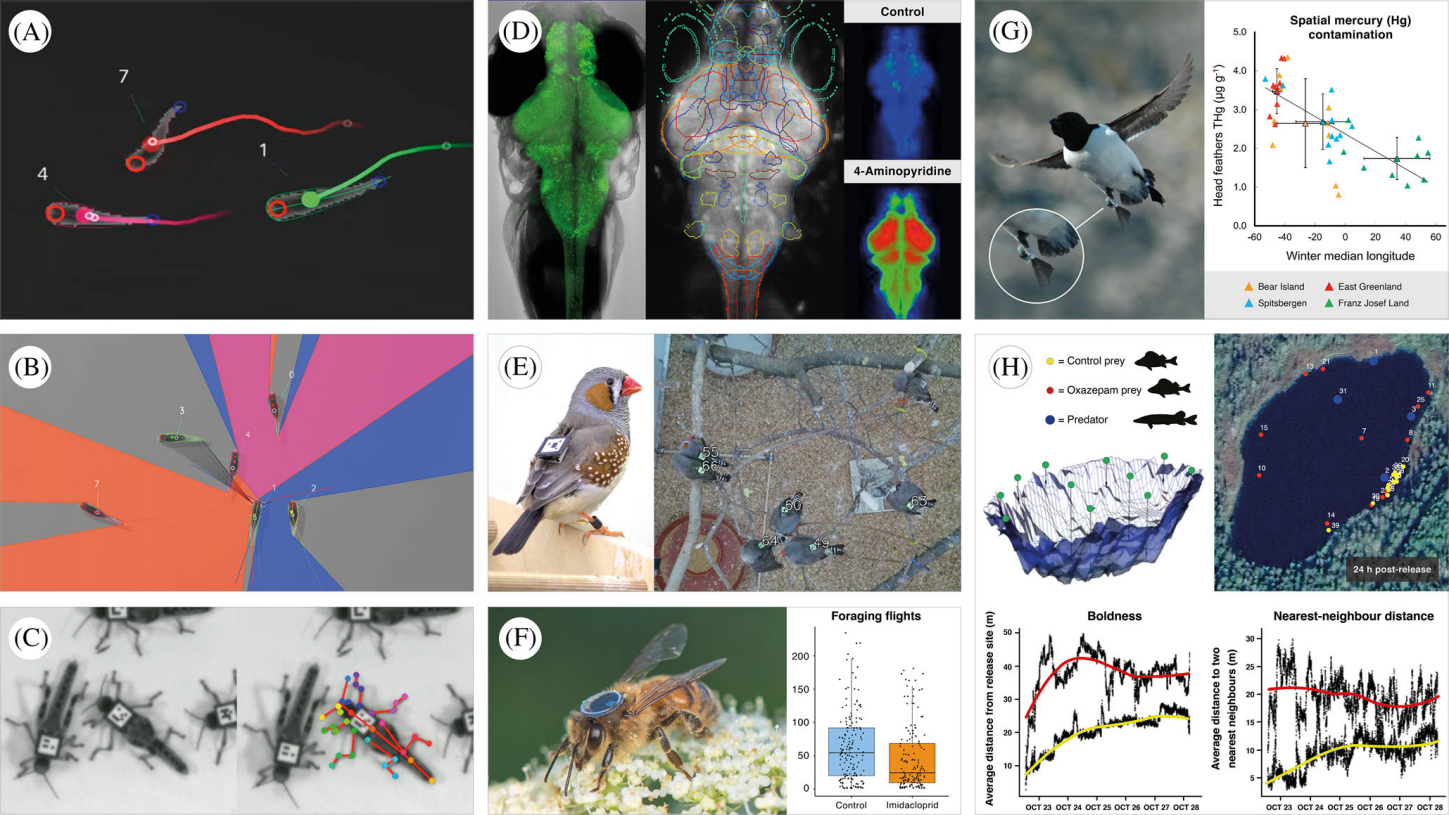
A wide variety of tools and technologies are now available to study potential impacts of contaminants on animal behaviour. Automated systems allow for (A) simultaneous tracking of large numbers of individuals (TRex; Walter & Couzin, 2021), and quantification of traits beyond movement, including (B) 2D visual fields (TRex; Walter & Couzin, 2021) and (C) posture analysis (DeepPoseKit; Graving *et al*., 2019). New technologies are also increasing mechanistic understanding in behavioural ecotoxicology, such as (D) functional neuroimaging with genetically encoded calcium sensors, used to quantify whole‐brain drug activity in larval fish (Winter *et al*., 2017). Further, innovative approaches enable tracking individuals in semi‐field and field settings: (E) automated barcode systems (Alarcón‐Nieto *et al*., 2018); (F) radio‐frequency identification (RFID) tagging, employed to reveal lifetime reductions in neonicotinoid‐exposed honey bee foraging (Colin *et al*., 2019); (G) Global Location Sensors (GLS), deployed to study spatial and seasonal mercury contamination of migratory little auks (*Alle alle*; Renedo *et al*., 2020); and (H) high‐resolution acoustic telemetry, used in a whole‐lake experiment to demonstrate reduced anxiety in prey fish exposed to pharmaceutical pollution (Klaminder *et al*., 2016). Image credits: A and B, T. Walter; C, J.M. Graving; D, M.J. Winter; E, G. Alarcón‐Nieto (left) and D. Farine (right); F, T. Colin; G, J. Fort; and H, J. Klaminder.

Here, we provide an overview of recent methodological and technological innovations that allow for the possible effects of contaminant exposure on organismal behaviour to be investigated at a greater resolution and scale than ever before (Fig. 2). See the online Supporting Information for a comprehensive overview of suppliers and resources for laboratory software and hardware (Tables S1 and S2, respectively), and field software and hardware (Tables S3 and S4).

### (1) Laboratory‐based experimental innovations

There is now a tremendous variety of laboratory software and hardware options available to ecotoxicologists for capturing potential behavioural responses to contaminant exposure. For instance, cameras with high frame rates offer the temporal resolution to track behaviours that were previously not possible to capture. This includes recording high‐speed locomotion on land (e.g. walking parameters of insects; Pfeffer *et al*., 2019), in water (e.g. propulsion in swimming fish; Johansen *et al*., 2017), and in the air (e.g. honeybee flight paths; Guiraud, Roper & Chittka, 2018), as well as feeding performance of predators (Rossoni & Niven, 2020), and escape performance of prey (e.g. Jonsson *et al*., 2019). Similarly, cameras with wide spectral‐ or thermal‐sensing capabilities enable researchers to record behaviours in the dark (e.g. Mitchell & Clarke, 2019), while multi‐camera systems track animal movement in three‐dimensional space (Macrì *et al*., 2017). Regarding high‐throughput approaches, lab‐on‐a‐chip devices, which make use of the changes in laminar flow within a chamber, have been developed for an array of aquatic organisms (Campana & Wlodkowic, 2018). These small chips are versatile to a variety of sensors and can measure behaviour following or during chemical exposure, in binary conditions as well as complex chemical gradients (Campana & Wlodkowic, 2018).

In addition to monitoring behaviours of pre‐exposed organisms or those exposed in real time, many new technologies have the ability to stimulate behaviours using light, vibration/noise, or electrical signals (discussed in Ågerstrand *et al*., 2020). This allows for the measurement of behavioural traits such as reaction time and speed, turning angles, space use, and predator avoidance. More sophisticated technologies are also being adapted to stimulate social, cognitive, and learning behaviours. For instance, state‐of‐the‐art robots can now be used to interact with fish in real time to study the underpinnings of social and anxiety‐related behaviours (e.g. Polverino *et al*., 2019), and even reveal the long‐lasting consequences of brief behavioural alterations on the life‐history, reproduction, and ecological success of targeted animals (Polverino *et al*., 2022). Indeed, despite being underexploited in behavioural ecotoxicology to date, bioinspired robots offer an autonomous, customisable, and repeatable approach that overcomes challenges associated with more traditional methods, in which live stimuli are an extra source of variation, although at the cost of some degree of biological and ecological realism. In addition to visual stimuli, new approaches also allow for increasingly sophisticated analysis of behavioural responses to chemical signals. For example, wind‐tunnel assays are allowing researchers to determine the preference of pollinating insects for non‐altered *versus* ozone‐contaminated floral blends (Cook *et al*., 2020), while two‐current choice flumes are increasingly used to examine chemosensory function in aquatic species (see Jutfelt *et al*., 2017), including attraction towards or avoidance of contaminants (Horký *et al*., 2021).

In parallel, automated visual tracking is fast becoming an indispensable tool for quantifying animal behaviour (reviewed in Henry & Wlodkowic, 2020; Panadeiro *et al*., 2021). There is now a wide variety of programs available for tracking animal movement, including a number of recently developed open‐source systems, such as TRex (Fig. 2A, Walter & Couzin, 2021) and ToxTrac (Rodriguez *et al*., 2018). Both of these systems are fast, powerful, user‐friendly, and versatile across a wide range of model species. Furthermore, these programs represent a major advance given their capacity to track large groups of animals simultaneously, for example ≤1000 individuals using TRex, including deep‐learning‐based visual identification of up to 100 unmarked individuals (Walter & Couzin, 2021). This provides an unparalleled opportunity to study the potential effects of contaminants on complex social behaviours in animal groups, such as dominance hierarchies and collective motion. Moreover, although distinctly underutilised in ecotoxicology to date, newly available software packages [e.g. DeepPoseKit (Graving *et al*., 2019); TRex (Walter & Couzin, 2021)] also allow for the quantification of an array of behavioural measures in addition to positional data, such as stereotyped movements (e.g. courtship displays), visual field (Fig. 2B), and posture analysis (e.g. body shape, head/tail position; Fig. 2C). As alluded to above, the integration of artificial intelligence and machine learning algorithms into these systems continues to power a revolution in motion‐tracking technology, including using deep learning to identify specific animals, determine posture, and even detect behaviours that were previously invisible to human observers (discussed in Graving *et al*., 2019).

From a mechanistic perspective, an increasing number of methods in molecular neurotoxicology are now available for uncovering drivers of pollutant‐induced behavioural changes (reviewed in Bownik & Wlodkowic, 2021). These methods range from transcriptomics (i.e. gene transcript analysis) to proteomics (i.e. analysis of proteins) and enzymatic assays, metabolomics (i.e. analysis of chemical processes involving metabolites in cells and tissues), and examination of sub‐cellular processes such as programmed cell death (Bownik & Wlodkowic, 2021). An important recent advance that promises to increase mechanistic understanding in behavioural ecotoxicology is large‐scale neurotransmitter profiling, enabled by the above‐mentioned rapid developments in analytical chemistry (see Section II.1). Here, an array of neurotransmitters can be monitored, as well as their precursors and degradation products, across different brain regions, providing a direct mechanistic link between pollutant‐induced behavioural changes and altered neuronal transmission (Mayol‐Cabré *et al*., 2020). For example, pioneering research harnessing neurotransmitter profiling recently demonstrated direct effects of the pervasive herbicide glyphosate on fish monoaminergic systems, with resulting effects on oxidative stress and anxiety‐related behaviours (Faria *et al*., 2021). Furthermore, significant recent breakthroughs mean that we can now dissect neural mechanisms for chemically induced brain activation and disruption using genetically encoded (bio)sensor systems coupled with high‐speed fluorescence light‐sheet microscopy (LSM; Winter *et al*., 2017; Fig. 2D). Illustrating this, transgenic zebrafish (*Danio rerio*) with pan‐neuronal expression of the calcium indicator GcaMP6, coupled with LSM, allow for functional imaging with sub‐cellular resolution across the whole brain, which has been applied to uncover mechanisms associated with behavioural responses to drug treatments (Winter *et al*., 2021).

### (2) Scaling up: experimental advances in the field

When assessing possible impacts of contaminants on behaviour, a holistic approach is ideal, that is studies relating mechanistic explanations to experiments spanning laboratory, semi‐field, and field contexts. While laboratory assays provide valuable insights in a standardised setting and can be important for validating field observations (both of which are fundamental to ecotoxicity studies to support various tiers of chemical risk assessments), semi‐field‐ and field‐based ecotoxicology is crucial for understanding the potential impacts of contaminants in large‐scale, complex, and dynamic natural systems. Encouragingly, experimental and technological innovations continue to emerge that allow for effects of chemical pollution on animal behaviour to be measured in the field with ever‐greater efficiency and accuracy. This includes advances in remote‐sensing technology, which have revolutionised our capacity to gather high‐resolution behavioural and physiological data from animals in the wild (Smith & Pinter‐Wollman, 2021; Nathan *et al*., 2022). Cheaper, smaller, and increasingly more capable transmitters and sensors allow researchers to tag and track animals over large spatial and temporal scales (Smith & Pinter‐Wollman, 2021; Nathan *et al*., 2022). For example, automated barcode systems enable whole‐group or even whole‐population tracking over months or potentially years (Alarcón‐Nieto *et al*., 2018; Fig. 2E). Meanwhile, radio‐frequency identification (RFID) tagging has revealed changes in the lifetime foraging behaviour of free‐ranging honeybees exposed to a neonicotinoid pesticide (Colin *et al*., 2019; Fig. 2F), and Global Location Sensors (GLS) have been used to study spatial and seasonal trends of mercury contamination in seabirds from Arctic breeding colonies (Renedo *et al*., 2020; Fig. 2G).

Perhaps the most transformative use of remote‐sensing technology can be found in aquatic environments, where tools such as acoustic telemetry are leapfrogging our understanding of the behaviour and ecology of aquatic organisms by enabling detailed spatial monitoring of individuals over whole freshwater systems (e.g. rivers and lakes; Fig. 2H) and even oceans (reviewed in Hellström *et al*., 2016b). Data previously difficult or impossible to obtain from the field, such as survival, schooling behaviour, home ranges, diel activity patterns, and predator–prey interactions can now be investigated in the wild at a level of detail comparable to that of laboratory studies (Hellström *et al*., 2016b). Further, we anticipate that future research pairing acoustic telemetry with newly developed methods of remote contaminant exposure (e.g. slow‐release implants; McCallum *et al*., 2019) and/or spatial contaminant modelling (Fonseca *et al*., 2020) will provide valuable insights into the real‐world behavioural impacts of chemical pollution. Parallel to the progress made in animal tracking technology, the ‘golden age of bio‐logging’ is providing an ever‐expanding variety of small physiological and behavioural sensors that can record everything from an animal's heart rate, body temperature, and acceleration, to detailed data on foraging and spawning behaviour (reviewed in Whitford & Klimley, 2019). In combination, these advances in remote‐sensing technology provide a powerful, yet largely unexplored, experimental toolkit to study impacts of pollution on behavioural and ecological processes. Harnessing these novel approaches will also enable research attempting to establish a causal chain from pollutant‐induced changes in individual‐ and group‐level behavioural variation to population responses and ecosystem change. Still, given the complexity of natural environments, it is important to note that careful experimental design (including the use of suitable controls), and appropriate statistical and biological interpretation of collected data, will be critical to the validity and reliability of such studies.

## V IMPROVING STATISTICAL SOPHISTICATION

Historically, ecotoxicology experiments for use in chemical risk assessment follow strict regulatory guidelines (Chapman *et al*., 1996). These guidelines have generally employed simple experimental designs and straightforward statistical analyses to maximise external validity and ensure protocols are broadly accomplishable by researchers (Chapman *et al*., 1996). However, ever‐increasing environmental and ecological complexity is being incorporated into behavioural ecotoxicology studies (see Sections II and III, respectively), and new technologies have changed the nature of the data extracted from these experiments (see Section IV). As a result, various statistical techniques conventionally used in ecotoxicology may not always be appropriate for use in modern behavioural ecotoxicology.

### (1) Embracing mixed modelling to address biological complexity

Biological data, and particularly behavioural endpoints, are often inherently variable, unbalanced (i.e. unequal sample sizes for different classes), non‐normally distributed, and highly structured (i.e. containing groups of non‐independent units). Such data sets require statistical approaches that are capable of appropriately partitioning and quantifying behavioural variation, while also considering the hierarchical nature of the data (i.e. to avoid pseudoreplication and/or to deal with non‐independence). One increasingly popular approach is mixed‐effects modelling (i.e. multilevel or hierarchical modelling) of either normally distributed response variables (linear mixed model, LMM) or non‐normally distributed response variables, such as count, binary or proportion data (generalised linear mixed models, GLMMs). Mixed‐effects models are a sophisticated statistical tool that can be applied to analyse data that have both fixed effects (e.g. experimental treatment, sex) and one or more clustering (random) factors (e.g. individual ID, treatment replicate) (Bolker *et al*., 2009). Mixed models can therefore account for, and estimate, the variation contributed by hierarchical structures in the data. A common example of this is the use of mixed models to account for multiple observations made on the same individual, where individual IDs are included in the random structure of the model as random intercepts to account for, and estimate, the amount of variation within and among individuals. This capability has meant that mixed models are now widely used in behavioural ecology and evolutionary biology to partition the variance across response variables both within and across grouping factors, and reveal effects occurring at each level (Dingemanse & Dochtermann, 2013). As previously discussed in this review, inferring the magnitude of variation within and among groups can be highly informative in the context of understanding responses to chemical pollution, which is now made possible *via* mixed‐modelling approaches, including using movement data in the wild (Hertel *et al*. 2020). Mixed models can also accommodate multiple response variables simultaneously (i.e. multivariate mixed models), allowing for the direct estimation of both within‐ and between‐individual phenotypic (co)variances. Further, particularly relevant for complex experimental designs, data‐reduction techniques (e.g. principal component analysis) can be used in combination with mixed modelling to collapse multiple related variables into fewer uncorrelated predictors in order to avoid over‐parameterisation. Fortunately, many publications on mixed models include detailed step‐by‐step guides on applying such statistical approaches to answer specific biological questions (summarised in Table S5).

### (2) Harnessing meta‐analytic approaches

We would also like to highlight the scarcity of meta‐analytic studies in the field of behavioural ecotoxicology. This is surprising given that sufficient data exist addressing behavioural effects of chemical pollutants to answer targeted meta‐analytic hypotheses/questions. Beyond this general call for meta‐analyses, we also suggest that behavioural ecotoxicologists draw from the recent advances in meta‐analytic methods developed in the fields of ecology and evolution (Nakagawa *et al*., 2017). For example, future meta‐analyses could focus on the impacts of chemical pollution on behavioural variance (i.e. total phenotypic variance of unexposed *versus* exposed populations), in parallel with a more ‘traditional’ mean‐focused meta‐analysis (Nakagawa *et al*., 2015). More specifically, in addition to comparing the means across different treatment groups of interest using standardised metrics such as Cohen's *d*, Hedges' *g*, or the response ratio, the natural logarithm of the ratio between the coefficients of variation (lnCVR) can also be extracted, allowing for meta‐analytical comparisons of differences between the variability in groups (for a detailed description, see Nakagawa *et al*., 2015). In addition, for broad research questions in behavioural ecotoxicology, systematic mapping will be beneficial to identify areas that require more research and those that are ready for meta‐analytic synthesis (see James, Randall & Haddaway, 2016), despite being currently underused in ecotoxicology (Wolffe *et al*., 2019). Further, to gain a more nuanced understanding of the structure of a research topic, a novel meta‐analytic technique called ‘research weaving’ could be used, which combines systematic mapping and bibliometrics (see Nakagawa *et al*., 2019).

## VI CONCLUSIONS

1. Behavioural studies represent a sensitive, powerful, and ecologically meaningful approach for assessing the effects of environmental contaminants.
2. As research in this area continues to grow and increase in complexity, we emphasise the importance of following basic principles of sound ecotoxicology wherever possible (Harris *et al*., 2014), for example using appropriate exposure route(s), testing a suitable number and span of exposure treatments, and quantifying exposure concentrations. Although not all principles apply to all studies, and certain principles can be challenging or impossible to implement in some cases (e.g. in large‐scale or long‐term studies), they are especially vital if researchers intend their work to be translationally applicable to chemicals risk assessment and/or regulation (Ågerstrand *et al*., 2020).
3. We encourage researchers working within behavioural ecotoxicology and related disciplines to explore the rich toolkit that is now available to propel our understanding of how chemical pollution impacts wildlife living in a rapidly changing world.

## Supporting information


**Table S1.** Software available to be incorporated into behavioural ecotoxicology research in the laboratory.
**Table S2.** Hardware available to be incorporated into behavioural ecotoxicology research in the laboratory.
**Table S3.** Software available to be incorporated into behavioural ecotoxicology research in the field.
**Table S4.** Hardware available to be incorporated into behavioural ecotoxicology research in the field.
**Table S5.** (a) A brief overview of resources available for implementing mixed modelling approaches in behavioural ecotoxicology, and (b) examples of specific research questions and resources for testing potential effects of contaminant exposure on behavioural variation at the individual level.
