# Supplementary material for: Frontiers in quantifying wildlife behavioural responses to chemical pollution
Source: Biol Rev Camb Philos Soc. 2022 Mar 1;97(4):1346–64. doi: 10.1111/brv.12844 (PMC9543409; doi:10.1111/brv.12844)
Supplement: Supplementary file 1 — Table S1. Software available to be incorporated into behavioural ecotoxicology research in the laboratory. Table S2. Hardware available to be incorporated into behavioural ecotoxicology research in the laboratory. Table S3. Software available to be incorporated into behavioural ecotoxicology research in the field. Table S4. Hardware available to be incorporated into behavioural ecotoxicology research in the field. Table S5. (a) A brief overview of resources available for implementing mixed modelling approaches in behavioural ecotoxicology, and (b) examples of specific research questions and resources for testing potential effects of contaminant exposure on behavioural variation at the individual level. [file BRV-97-1346-s001.docx]

**SUPPORTING INFORMATION**

**Frontiers in quantifying wildlife behavioural responses to chemical pollution**

Michael G. Bertram*, Jake M. Martin, Erin S. McCallum, Lesley A. Alton, Jack A. Brand, Bryan W. Brooks, Daniel Cerveny, Jerker Fick, Alex T. Ford, Gustav Hellström, Marcus Michelangeli, Shinichi Nakagawa, Giovanni Polverino, Minna Saaristo, Andrew Sih, Hung Tan, Charles R. Tyler, Bob B.M. Wong, Tomas Brodin

* Author for correspondence:

Michael G. Bertram

Department of Wildlife, Fish, and Environmental Studies

Swedish University of Agricultural Sciences

Umeå SE-907 36, Sweden, michael.bertram@slu.se

**CONTENTS**

**Table S1.** Software available to be incorporated into behavioural ecotoxicology research in the laboratory.

**Table S2.** Hardware available to be incorporated into behavioural ecotoxicology research in the laboratory.

**Table S3.** Software available to be incorporated into behavioural ecotoxicology research in the field.

**Table S4.** Hardware available to be incorporated into behavioural ecotoxicology research in the field.

**Table S5.** (a) A brief overview of resources available for implementing mixed modelling approaches in behavioural ecotoxicology, and (b) examples of specific research questions and resources for testing potential effects of contaminant exposure on behavioural variation at the individual level.

**Table S1.** Software available to be incorporated into behavioural ecotoxicology research in the laboratory.

| **Name** | **Supplier and link** | **Cost*** | **Description** | **Useful references** |
| --- | --- | --- | --- | --- |
| **Video tracking** |  |  |  |  |
| Ctrax | California Institute of Technology,  http://ctrax.sourceforge.net/ | Free | Automated animal tracking. Optimised for tracking fruit flies (*Drosophila melanogaster*) on a uniform 2D surface. | Branson *et al.* (2009) |
| DANNCE | Timothy W. Dunn/Jesse D. Marshall,  https://github.com/spoonsso/dannce | Free | Automated animal tracking and pose estimation in 3D using neural networks. Allows for markerless, video-based landmark tracking of freely behaving animals. Available as an open-source python package. | Dunn *et al.* (2021) |
| DeepLabCut | Mathis Labs,  http://www.deeplabcut.org/ | Free | Automated animal tracking using neural networks. Designed for animal pose estimation. | Nath *et al.* (2019) |
| DeepPoseKit | Max Planck Institute of Animal Behavior,  https://github.com/jgraving/DeepPoseKit | Free | Automated animal tracking using neural networks. Designed for animal pose estimation. | Graving *et al.* (2019) |
| FastTrack | Benjamin Gallois,  http://www.fasttrack.sh/ | Free | Automated animal tracking. Ability to track multiple individuals simultaneously. Capable of maintaining individual identities across video recordings. Allows for manual correction of tracking errors. | Gallois & Candelier (2021) |
| idTracker | Cajal Institute,  http://www.idtracker.es/home | Free | Automated animal tracking. Requires MATLAB Compiler Runtime. Optimised for maintaining individual identities while tracking multiple individuals simultaneously. | Pérez-Escudero *et al.* (2014) |
| idtracker.ai | Champalimaud Foundation,  https://idtrackerai.readthedocs.io/en/latest/ | Free | Automated animal tracking using neural networks. Designed for maintaining individual identities while tracking multiple individuals simultaneously (up to 100). | Romero-Ferrero *et al.* (2019) |
| Image J/Fiji | National Institutes of Health,  https://imagej.nih.gov/ij/  https://imagej.net/software/fiji/ | Free | Automated and manual animal tracking available with additional plugins (e.g. MTrackJ, wrMTrck). | Meijering *et al.* (2012); Schneider *et al.* (2012); Nussbaum-Krammer *et al.* (2015) |
| pirecorder | Jolle Jolles, https://github.com/JolleJolles/pirecorder | Free | A Python package for controlling video recordings made with Raspberry Pi. Main functionalities include the control and scheduling of video-recordings, ability to optimise camera configuration for video and image collection, and automatic file naming and sorting. | Jolles (2020) |
| SLEAP | Murthy Lab/Princeton University,  https://sleap.ai/ | Free | Automated animal tracking using neural networks. Designed for multi-animal pose estimation. | Pereira *et al.* (2019, 2020) |
| Tierpsy Tracker | Andre Brown, https://github.com/Tierpsy/tierpsy-tracker | Free | Automated animal tracking. Optimised for simultaneously tracking multiple individuals of the roundworm, *C. elegans*. Can extract postural information and has been used to track the movement of fish and fruit fly larvae. | Javer *et al.* (2018) |
| ToxTrac | Umeå University,  https://sourceforge.net/projects/toxtrac/ | Free | Automated animal tracking. Main capabilities include fast processing speed, simultaneous tracking of multiple individuals, and analysis tools. | Rodriguez *et al.* (2018) |
| Tracktor | Max Planck Institute of Animal Behaviour/ University of Konstanz, https://github.com/vivekhsridhar/tracktor | Free | Automated animal tracking. Code-based program designed for single-object tracking in noisy environments or multi-individual tracking in uniform environments. | Sridhar *et al.* (2019) |
| TRex | Tristan Walter/Iain D. Couzin/Max Planck Institute of Animal Behaviour,  https://trex.run/ | Free | Automated animal tracking using neural networks. Contains tools for pose estimation and visual field reconstruction. Capable of tracking hundreds of individuals simultaneously. | Walter & Couzin (2021) |
| UMATracker | Osamu Yamanaka/Rito Takeuchi,  http://ymnk13.github.io/UMATracker/ | Free | Automated animal tracking. Contains tools for image pre-processing and manual tracking-error correction. | Yamanaka & Takeuchi (2018) |
| WormPose | Greg J. Stephens, https://github.com/iteal/wormpose | Free | Automated animal tracking using neural networks. Designed for 2D pose estimation of the roundworm, *C. elegans*. Optimised for estimating postures of complex, self-occluded, coiled shapes. | Hebert *et al.* (2021) |
| EthoVision XT | Noldus Information Technology B.V.,  https://www.noldus.com/ethovision-xt | $$$$ | Automated animal tracking. Capabilities include tracking and analysis of behaviour, movement, and activity, as well as manual event-logging capabilities. Price depends on location and functions included. | Spink *et al.* (2001) |
| LoliTrack | Loligo Systems,  https://loligosystems.com/lolitrack-version-5-video-tracking-and-behavior-analysis-software | $$$ | Automated animal tracking. Capabilities include both 2D and 3D tracking, ability to track multiple individuals simultaneously, and analysis tools. | Loligo-Systems (in press) |
| VideoTrack | ViewPoint Behavior Technology,  http://www.viewpoint.fr/en/p/software/videotrack | $$$$ | Automated animal tracking. Optimised for tracking rodent behaviour. Capabilities include simultaneous tracking in multiple arenas, and specific templates for several behavioural assays (e.g. elevated plus maze, Y maze, Morris water maze). |  |
| ZebraLab | ViewPoint Behavior Technology,  http://www.viewpoint.fr/en/p/software/zebralab-zebrafish-behavior-screening | $$$$$ | Automated animal tracking. Optimised for tracking zebrafish behaviour. Able to track multiple larvae simultaneously in multi-well plates, as well as adult fish both individually and in shoals. |  |
| **Event logging** |  |  |  |  |
| BORIS | Olivier Friard/ Marco Gamba/University of Torino,  https://www.boris.unito.it/ | Free | Event-recording software. Features include event-logging for recordings or live observations, and the ability to define an unlimited number of behaviours and subjects. | Friard & Gamba (2016) |
| EthoWatcher | Federal University of Santa Catarina,  https://ethowatcher.paginas.ufsc.br/ | Free | Event-recording software. Also includes animal tracking capabilities. | Crispim Junior *et al.* (2012) |
| JWatcher | Blumstein Lab/ University of California Los Angeles,  http://www.jwatcher.ucla.edu/ | Free | Event-recording software. Designed to score a single subject’s behaviour in a continuous observation. | Blumstein & Daniel (2007) |
| BehaviorCloud | BehaviorCloud,  https://www.behaviorcloud.com/ | $$$/year | Browser-based program that includes event-recording and tracking capabilities. Also includes data storage and analysis tools, and the ability to share and collaborate on projects with multiple users simultaneously. Price depends on subscription length. | BehaviorCloud (in press) |
| LabWatcher | ViewPoint Behavior Technology,  http://www.viewpoint.fr/app.php/en/p/software/labwatcher | $$ | Event-recording software. Capabilities include scoring from pre-recorded or live videos, and analysis tools. |  |
| Loopy | Loopbio,  http://loopbio.com/loopy/ | $$/year | Browser-based program that includes event-recording capabilities. Allows the user to upload, organise, and share video files with collaborators *via* an online platform. Both 2D and 3D tracking, deep learning, and pose estimation tools available with more advanced subscription. |  |
| The Observer XT | Noldus Information Technology B.V.,  https://www.noldus.com/observer-xt-animal | $$$$ | Event-recording software. Includes analysis capabilities and can be integrated with data from other recording devices (e.g. physiological data). Price depends on location and functions included. | Zimmerman *et al.* (2009) |
| ZooMonitor | Lincoln Park Zoo,  https://zoomonitor.org/home | $/year | Browser-based application that includes event-recording capabilities. Can be used on desktop computer or tablet devices. Allows the user to create different projects and analyse results through built-in data visualization tools. Users can also export data for manual analysis. | Wark *et al.* (2019) |

* Software prices: Free (US$0), $ (<US$500), $$ (<US$1000), $$$ (<US$5000), $$$$ (<US$10,000), and $$$$$ (>US$10,000). Pricing is approximate and based on currency conversion as of 10-01-2022.

**Table S2.** Hardware available to be incorporated into behavioural ecotoxicology research in the laboratory.

| **Name** | **Supplier and link** | **Cost^†^** | **Description** | **Useful references** |
| --- | --- | --- | --- | --- |
| **Experimental arena** |  |  |  |  |
| DanioVision | Noldus Information Technology B.V.,  https://www.noldus.com/daniovision/observation-chamber | $$$$$ | High-throughput monitoring arena for analysis of larval fish or small animal behaviour. Allows control of physical parameters. Ability to track in the dark with infrared-sensitive camera. Includes EthoVision XT software, experimental chamber, temperature-control unit, and a computer. |  |
| Kastl | Loopbio,  http://loopbio.com/kastl/ | Price available upon request | Custom-built experimental setup. Incorporates the Motif recording system in a versatile behavioural arena. Designed for diverse, high-throughput behavioural experiments with several model species. Integrates with the Loopy behavioural analysis software from Loopbio. |  |
| ToxmateLAB | ViewPoint Behavior Technology,  http://www.viewpoint.fr/en/p/equipment/toxmatelab | $$$$$ | Behavioural arena designed to track and record behavioural data of small animals. Allows the delivery of aqueous or gaseous toxicants to measure their impact on behaviour in real time. |  |
| Zantiks LT | Zantiks Ltd,  https://zantiks.com/index.php?p=products/zantiks-lt | $$$$ | Experimental chamber designed for analysis of rodent and small fish behaviour. Includes enclosure, computer, cameras, infrared lighting, and relevant software for running behavioural experiments. Allows for the control of arena illumination, delivery of solid or liquid food rewards, and the display of visual stimuli using an inbuilt screen. |  |
| ZebraBox | ViewPoint Behavior Technology,  http://www.viewpoint.fr/en/p/equipment/zebrabox-for-embryos-or-larvae | $$$$$ | High-throughput monitoring enclosure for tracking zebrafish larvae and embryos. Capable of analysing multiple individual zebrafish larvae (up to 96 individuals) in multi-well plates. Includes several lighting options (e.g. infrared) and a water flow-through system. A number of add-ons are available, including the ability to deliver electrical signals. |  |
| ZebraCube | ViewPoint Behavior Technology,  http://www.viewpoint.fr/en/p/equipment/zebracube-adult-zebrafish-behavior-analysis | $$$$$ | Behavioural arena optimised for testing adult zebrafish behaviour. Integrates with ZebraLab software. Allows for control of physical parameters. Add-ons are available (e.g. temperature control, electrical signals, high-speed camera). |  |
| **Virtual reality** |  |  |  |  |
| FishVR | Loopbio,  http://loopbio.com/fishvr/ | Price available upon request | Visual virtual reality system for free-swimming fish. Custom-built depending on user requirements. Allows the design of 3D virtual environments (e.g. virtual backgrounds and arenas) and stimuli (e.g. virtual objects within the environment). Includes the Kastl and Motif multi-camera system (i.e. computer, projector, arena, cameras), and relevant software for running experimental trials. Installation and training can also be included. | Stowers *et al.* (2017) |
| FlyCave and MouseVR (non-commercial) | Not commercially available as a whole system.  Individual parts available from respective suppliers. | Not commercially available | Virtual reality systems for freely moving flies and mice, respectively. | Stowers *et al.* (2017) |
| **Cameras and recording systems** |  |  |  |  |
| FLIR T530sc | FLIR Systems, Inc.,  https://www.flir.com/products/t530/ | $$$$$ | High-resolution thermal imaging camera. Thermal measuring range of −20 to 650 °C ± 2 °C. Price is for base model with 24° lens. |  |
| GoPro Hero8 Black | GoPro, Inc.,  https://gopro.com/en/de/shop/cameras/hero8-black/CHDHX-801-master.html | $ | High-resolution (4K) and relatively high-speed (up to 240 fps) waterproof camera. Includes image-stabilisation tools. Add-ons are available (e.g. lighting, mounts, housing). Price varies by location. |  |
| InfReC R550 Series | Nippon Avionics Co.,  http://www.infrared.avio.co.jp/en/products/ir-thermo/lineup/r550/index.html | $$$$$ | High-resolution infrared thermal imaging camera. Thermal measurement capabilities range from −40 to 650 °C ± 1 °C. Price is for the base model. |  |
| Motif | Loopbio,  http://loopbio.com/recording/ | Price available upon request | High-quality and high-speed video-recording system (i.e. camera, computer, and software). Can be used in both laboratory and field settings. Supports single- and multiple-camera recordings. |  |
| MotionBLITZ Eo*Sens* Cube7 | MIKROTRON,  https://mikrotron.de/en/products/high-speed-recording-cameras/motionblitzr-cube.html | $$$$$ | High-speed and high-resolution camera. Can record at up to 523 fps at 1696 × 1710-pixel resolution. Additional features are available (e.g. longer recording times, camera mounts). Price is for the base model. |  |
| Sony FDR-AX43 | Sony,  https://www.sony.com/tz/electronics/handycam-camcorders/fdr-ax43 | $$ | Versatile high-resolution (4K) and relatively fast-speed camera. Includes image stabilisation features. |  |
| **Robotics** |  |  |  |  |
| Bioinspired robotic fish (non-commercial) | Not commercially available as a whole system.  Individual parts available from respective suppliers. | Not commercially available | Bioinspired robotic fish used in behavioural research to investigate interactions between focal individuals and artificial con- and/or heterospecifics. | Kopman *et al.* (2013); Kopman & Porfiri (2013); Spinello *et al.* (2013); Polverino *et al.* (2019, 2022) |
| InsBot robot -  Insect-robot  (non-commercial) | Not commercially available as a whole system.  Individual parts available from respective suppliers. | Not commercially available | Bioinspired robotic insect used in behavioural research to investigate social behaviour of cockroach groups. | Colot *et al.* (2004); Asadpour *et al.* (2006); Halloy *et al.* (2007) |
| **Lab-on-a-chip** |  |  |  |  |
| GammarusChip  (non-commercial) | Not commercially available as a whole system.  Individual parts available from respective suppliers. | Not commercially available | GammarusChip is composed of a flow-through ‘chip’ system, holding a small volume of water. This system has the capability to deliver low concentrations of toxicants to a central chamber containing small invertebrates (e.g. amphipods). Camera systems then record basic behaviours (e.g. activity) to investigate how these behaviours might change after the introduction of toxicants. | Cartlidge *et al.* (2015) |
| **Computing** |  |  |  |  |
| Raspberry Pi microcomputers | Raspberry Pi,  https://www.raspberrypi.org/ | $ | Highly flexible, low-cost microcomputer. Capable of long-term image and/or video recording. Raspberry Pi devices have been used for a wide variety of applications, including home cage monitoring for animal husbandry, camera-traps, high-throughput behavioural recordings, and nest box monitoring. | Jolles (2021) |

† Hardware prices: $ (<US$500), $$ (<US$1000), $$$$ (<US$10,000), and $$$$$ (>US$10,000). Pricing is approximate and based on currency conversion as of 10-01-2022. Prices may also vary by region depending on shipping, clearance, and tax. Cost is provided for one unit (e.g. one experimental arena, one camera), of which multiple may be required.

**Table S3.** Software available to be incorporated into behavioural ecotoxicology research in the field.

| **Name** | **Supplier and link** | **Cost** | **Description** | **Useful references** |
| --- | --- | --- | --- | --- |
| **Deep learning frameworks** |  |  | A subfield of machine learning that involves the use of artificial neural networks to automatically generate predictive models by detecting patterns in data with or without supervised training. Useful for analysing extremely large and complex data sets that may otherwise be difficult to analyse manually. | Browning *et al.* (2018); Christin *et al.* (2019); Beyan & Browman (2020) |
| Tensorflow | Google Brain Team, https://www.tensorflow.org/ | Free | Open source machine learning framework. |  |
| Keras | François Chollet,  https://keras.io/ | Free | A deep learning interface built upon Tensorflow 2.0 to provide simpler application programming interfaces (APIs). |  |
| Pytorch | Adam Paszke/Sam Gross/Soumith Chintala/Gregory Chanan/Facebook’s AI Research lab, https://pytorch.org/ | Free | Open source machine learning framework. |  |
| H2O | H2O.ai,  https://www.h2o.ai/ | Free | Open source machine learning framework. |  |
| Apache MXNet | The Apache Software Foundation, https://mxnet.apache.org/ | Free | Open source machine learning framework. |  |
| Microsoft Cognitive Toolkit | Microsoft, https://docs.microsoft.com/en-us/cognitive-toolkit/ | Free | Open source toolkit for commercial-grade distributed deep learning. |  |
| Eclipse Deeplearning4j | Konduit/Eclipse Foundation, https://deeplearning4j.konduit.ai/ | Free | Commercial-grade, open source, distributed deep learning library written for Java and Scala. |  |
| Deep Learning Toolbox | Mathworks, https://www.mathworks.com/products/deep-learning.html | License costs vary | Deep learning framework for use with MATLAB. License costs dependent on purchaser’s circumstances. Requires MATLAB software. |  |
| **Animal tracking and monitoring software** |  |  | A wide variety of software options and R packages are available for analysing animal movement and monitoring data. For a comprehensive overview of available R packages, see Joo *et al.* (2020) and references cited therein. | Joo *et al.* (2020) |
| yaps | Henrik Baktoft, https://github.com/baktoft/yaps | Free | Open source R package created to estimate positions of fish and other aquatic animals tagged with acoustic transmitters. Alternative to closed source manufacturer-provided software. | Baktoft *et al.* (2017) |
| BEEtag | James Crall, https://github.com/jamescrall/BEEtag | Free | Open source software package in MATLAB designed for tracking uniquely identifiable visual markers. | Crall *et al.* (2015) |
| BeesBook | Benjamin Wild/David Dormagen/Leon Sixt/Adrian Zachariae/Sophie Zabel/Tim Landgraf | Not commercially available | A custom-made system for automatic tracking of all individuals in a bee colony, and decoding of communication dances. Full specifications of the recording setup and automatic recognition software are detailed in the relevant references. | Wario *et al.* (2015); Smith *et al.* (2021); Wild *et al.* (2021) |
| Detectron2  (superseding Mask R-CNN for PyTorch) | Facebook’s AI Research lab, https://github.com/facebookresearch/detectron2 | Free | Implements object-detection algorithms, powered by the PyTorch deep learning framework. Has been used to automate the analysis of fish abundance from video footage taken in the wild. | Ditria *et al.* (2020) |
| Mask R-CNN for TensorFlow/Keras | Matterport, https://github.com/matterport/Mask_RCNN | Free | Implementation of a Mask and Region-based Convolution Neural Network (Mask R-CNN) on Python 3, Keras, and TensorFlow deep learning frameworks for object detection and segmentation. Can be used to detect and estimate poses and trajectories of animals from video footage taken in the wild. | Francisco *et al.* (2020) |
| Deep active learning for camera trap images | Mohammad Sadegh Norouzzadeh/Dan Morris/Sara Beery/Neel Joshi/Nebojsa Jojic/Jeff Clune,  https://github.com/microsoft/CameraTraps/tree/norouzzadeh-et-al-2020/research/active_learning | Free | A method for automated species classification, monitoring, and counting from camera trap images. Uses a novel deep learning approach that minimises manual labour while maintaining accuracy. | Norouzzadeh *et al.* (2021) |
| Deep neural networks for camera trap imaging | Mohammad Sadegh Norouzzadeh/Anh Nguyen/Margaret Kosmala/Ali Swanson/Meredith Palmer/Craig Packer/Jeff Clune, https://github.com/Evolving-AI-Lab/deep_learning_for_camera_trap_images | Free | Deep neural network architectures used to automate information extraction from camera-trap imaging. Specifically, neural networks can be trained to detect images that contain animals, count animals, identify species, and characterise behaviours. | Norouzzadeh *et al.* (2018) |
| DeepPoseKit | Max Planck Institute of Animal Behavior, https://github.com/jgraving/DeepPoseKit | Free | Automated animal tracking using neural networks. Designed for animal pose estimation. | Graving *et al.* (2019) |
| Wild-ID | Douglas Thomas Bolger, https://envs.dartmouth.edu/people/douglas-thomas-bolger | Free | Open source software created to assist with pattern extraction and image matching of wild animals in photographic mark–recapture analysis. Works on the basis that individuals have patterns on their coat or skin that are sufficiently variable to discriminate among individuals within a population. | Bolger *et al.* (2012) |
| VTrack | Hamish A. Campbell/Matthew E. Watts/Ross G. Dwyer/Craig E. Franklin, https://github.com/RossDwyer/VTrack | Free | Open source R software package created to facilitate the assimilation, analysis, and synthesis of animal location data collected by Innovasea acoustic-tracking equipment. Provides database and visualisation capabilities, and functions to identify ecologically relevant events from tag detection and sensor data. | Campbell *et al.* (2012) |
| Argus | Brandon E. Jackson/Dennis J. Evangelista/Dylan D. Ray/Tyson L. Hedrick, http://argus.web.unc.edu/ | Free | A package of open source software with an easy-to-use interface designed for performing multi-camera 3D data acquisition using inexpensive consumer-grade cameras. | Jackson *et al.* (2016) |
| Behayve | Living Ocean (livingocean.org.au),  https://www.behayve.com/ | Refer to website | A smartphone application for recording animal behavioural observations and tracks in the field. Capable of recording GPS, compass, and elevation information. Ability to export data in formats compatible with a variety of Geographic Information Systems (GIS). |  |

**Table S4.** Hardware available to be incorporated into behavioural ecotoxicology research in the field.

| **Category** | **Relevant equipment** | **Example product, supplier, and link** | **Cost^§^** | **Description** | **Useful references** |
| --- | --- | --- | --- | --- | --- |
| **Aquatic animal tracking** |  |  |  |  | Hussey *et al.* (2015); Hays *et al.* (2016); Lennox *et al.* (2017); Nathan *et al.* (2022) |
| *Acoustic telemetry* |  |  |  | For short- to long-term, fine-scale movements in a spatially restricted area due to reliance on transmission to proximate receivers. | Donaldson *et al.* (2014); Haulsee *et al.* (2016); Bruneel *et al.* (2020); Griffin *et al.* (2020) |
|  | Acoustic transmitter tag | V7 69 kHz non-sensor coded transmitter, Innovasea Systems Inc.,  https://www.innovasea.com/ | $ | Emits acoustic waves carrying unique identification information that is communicated to a proximate acoustic receiver. Sizes vary to suit study species sizes, allowing for implantation or external attachment to an animal. |  |
|  | Fixed acoustic receiver | VR2Tx 69 kHz coded acoustic receiver, Innovasea Systems Inc.,  https://www.innovasea.com/ | $$$ | Receiver that decodes acoustic signals from transmitter tags. Typically fixed in an array covering locations of importance or interest. Can be individually submerged, connected underwater *via* cables, or attached to anchored or drifting buoys. |  |
|  | Portable acoustic receiver | VR100-300 mobile DSP tracking receiver, Innovasea Systems Inc.,  https://www.innovasea.com/ | $$$$ | Designed for mobile tracking: actively seeking out transmitter tags with a portable receiver, as opposed to fixed receivers that are locked in place to detect acoustic signals within their proximity. |  |
|  | Animal-borne transceiver | Vemco Mobile 69 kHz Transceiver (VMT), Innovasea Systems Inc.,  https://www.innovasea.com/ | $$$ | A hybrid transmitter and receiver externally attached to larger animals. Useful for studying intra- and interspecific interactions (e.g. predator–prey interactions). |  |
|  | Acoustic data storage tag | Acoustic data storage tag (ADST), Innovasea Systems Inc.,  https://www.innovasea.com/ | $$ | Device attached to an animal that both acoustically transmits and internally stores essential telemetry sensor data such as temperature and depth. Sensor data gathered throughout the life of the tag is accessible once retrieved. |  |
|  | Accelerometer tag | V13AP accelerometer tag with pressure sensor, Innovasea Systems Inc., https://www.innovasea.com/ | $$ | Device that is either implanted into or externally mounted onto an animal, which measures activity by transmitting 3D acceleration data as the animal moves within a receiver array. |  |
| *Satellite telemetry* |  |  |  | For long-term, fine- to coarse-scale movements over broad unrestricted distances. Limited to use on larger animals when compared to acoustic telemetry. | Dujon *et al.* (2014); Thomson *et al.* (2017) |
|  | Argos tag | SPOT and SPLASH10 series Argos tags, Wildlife Computers Inc.,  https://wildlifecomputers.com/ | $$$ | Versatile tags that transmit to the Argos satellite system for tracking horizontal and vertical movement. For use with animals that routinely or periodically spend time at the water surface or return to land. Animals must completely clear the water surface to transmit messages successfully to Argos satellites. |  |
|  | Fastloc GPS tag | SPLASH10-BF Basic Fastloc^®^ Argos tag, Wildlife Computers Inc.,  https://wildlifecomputers.com/ | $$$ | Satellite-transmitting tags that use Fastloc GPS technology, which allows for finer-scale and more accurate location tracking of animals. Fastloc GPS technology is useful in dynamic environments where satellite signals are only available for brief periods of time. Fastloc typically acquires tracking positions more frequently compared to Argos-only tags. |  |
|  | Pop-up satellite archival tag | MiniPAT Argos-linked pop-up archiving tag, Wildlife Computers Inc.,  https://wildlifecomputers.com/ | $$$ | For use with animals that do not breach the water surface and thus cannot typically be tracked with Argos or Fastloc GPS tags. Tags are attached externally, then archive data and are released once certain parameters are met. Upon release, tags float to the surface and transmit archived data to the Argos satellite network. |  |
| *Radio telemetry* |  |  |  | For short- to long-term, fine- to coarse-scale movements in a spatially restricted area. Identifies tagged animals through manual tracking or fixed stations that detect radio signals emitted by active transmitters. | Cooke *et al.* (2013) |
|  | VHF transmitter | F-series transmitters, Advanced Telemetry Systems, Inc.,  https://atstrack.com/ | $ | Very-high-frequency (VHF), battery-powered radio transmitter that can be attachment to or implanted in an animal. |  |
|  | VHF receiver and datalogger | R410, R2000, and R4500 series receivers, Advanced Telemetry Systems, Inc.,  https://atstrack.com/ | $$–$$$$ | Basic models only receive VHF transmissions, while higher-end models have an integrated GPS that logs location data. Requires additional antennae accessories to receive radio signals. |  |
| *Radio frequency identification (RFID)* |  |  |  | For short- to long-term, coarse-scale movements in a spatially restricted area. Identifies tagged animals through manual scanning or fixed automated receivers that scan passing individuals. Tags can be unpowered/passive and, therefore, long-lasting. | Cooke *et al.* (2013) |
|  | PIT tag | HPT9 tag, Biomark, Inc.,  https://www.biomark.com/ | $ | Passive unpowered tags that use magnetic induction to transmit a unique code to a receiver. Requires loading into a needle for implantation into an animal. |  |
|  | Implanter | MK25, Biomark, Inc.,  https://www.biomark.com/ | $ | Used to implant tags into animals. |  |
|  | Portable reader | HPR Plus, Biomark, Inc.,  https://www.biomark.com/ | $$$ | Portable reader coupled with an attached antenna for manual scanning and tracking. Largely limited to tracking in shallower systems. |  |
|  | Antenna systems | Cord antenna system, Biomark, Inc.,  https://www.biomark.com/ | $$$ | Includes a reader and antennae that can be deployed to create fixed stations that automatically read tags of passing individuals. Typically used in lotic systems or areas where animals congregate. |  |
| *Light-based geolocation / geolocation* |  |  |  | Also termed global location sensors (GLS). For tracking long-term, coarse-scale movements over broad distances. Uses observation of environmental data such as ambient light level, temperature, and depth (in the case of aquatic geolocation) along with corresponding reference data to determine an animal’s location and trajectory. |  |
|  | Archival non-transmitting tag | TDR-Mk9 and TDR10 series tags, Wildlife Computers Inc.,  https://wildlifecomputers.com/ | $$–$$$ | Records and archives positional data on-board the tag. Typically requires recovery of the tag to acquire data. Can be paired with a variety of sensors (e.g. accelerometers). |  |
| *Autonomous underwater vehicle* |  |  |  | A robot that travels underwater without requiring input from an operator. A variety of sensors can be affixed to these vehicles for research use. |  |
|  |  | Iver3 and Iver4 series AUV, L3Harris Technologies, Inc.,  https://www.l3harris.com/ | $$$$$ | When paired with acoustic telemetry, can be outfitted with receivers to autonomously track and follow aquatic animals tagged with an acoustic transmitter. | Lin *et al.* (2017) |
| **Terrestrial animal tracking** |  |  |  |  | Kays *et al.* (2015); Nathan *et al.* (2022) |
| *Satellite telemetry* |  |  |  | For long-term, fine- to coarse-scale movements over broad unrestricted distances. | Cheng *et al.* (2019); Sergio *et al.* (2019) |
|  | GPS-satellite tracker | Solar Argos/GPS PTT, Microwave Telemetry, Inc.,  https://www.microwavetelemetry.com/ | $$$ | Uses both GPS and Argos technology to provide both GPS positions (high accuracy) and doppler positions (larger margin of error) for both fine- and broad-scale tracking. |  |
|  | GPS/GSM trackers | GPS/GSM 20-70 transmitters, Microwave Telemetry, Inc.,  https://www.microwavetelemetry.com/ | $$$ | Uses the mobile data network to transfer GPS positional data. Frequent data collection and transmission. Ideal for tracking fine-scale movements and habitat use. Operates at a higher capacity, and has a more direct means of data transfer, compared to a satellite transmission system. |  |
|  | Argos tracker | Solar- or battery-powered PTT, Microwave Telemetry, Inc.,  https://www.microwavetelemetry.com/ | $$$ | Uses Argos satellite triangulation to provide doppler positions that can be categorised into location classes based on their estimated accuracy (<250 m to >1500 m radius). Less accurate than GPS tracking. |  |
| *Radio telemetry* |  |  |  | For short- to long-term, fine- to coarse-scale movements in a spatially restricted area. Identifies tagged animals through manual tracking or fixed stations that detect radio signals emitted by active transmitters. |  |
|  | ATLAS tag | ATLAS tag (in printed circuit board-form only), Minerva Center for Movement Ecology, https://www.tau.ac.il/~stoledo/tags/ | $–$$ | Real-time high-resolution movement tracking of many small animals simultaneously for relatively long durations at a comparatively low cost. However, still in the early stages of production and requires additional infrastructure at relatively high costs. Limited spatial coverage compared to GPS tracking. | Toledo *et al.* (2016, 2020); Weiser *et al.* (2016) |
|  | Motus | NanoTags, Lotek Wireless Inc. or LifeTag and PowerTag, Cellular Tracking Technologies,  see https://motus.org/ | $ | The Motus Wildlife Tracking System is an international network of researchers using coordinated, automated radio-telemetry arrays to study movements of small flying organisms. Details on how to join this network can be found at https://motus.org/ | Taylor *et al.* (2017) |
|  | VHF transmitter | M-series transmitters, Advanced Telemetry Systems, Inc.,  https://atstrack.com/ | $ | Very-high-frequency (VHF), battery-powered radio transmitter that can be attachment to or implanted in an animal. |  |
|  | VHF receiver and datalogger | R410, R2000, and R4500 series receivers, Advanced Telemetry Systems, Inc.,  https://atstrack.com/ | $$–$$$$ | Basic models only receive VHF transmissions, while higher-end models have an integrated GPS that logs location data. Requires additional antennae accessories to receive radio signals. |  |
| *Light-based geolocation / geolocation* |  |  |  | Also termed global location sensors (GLS). For tracking long-term, coarse-scale movements over broad distances. Uses observation of environmental data such as ambient light level, temperature, and depth (in the case of aquatic geolocation) along with corresponding reference data to determine an animal’s location and trajectory. |  |
|  | Archival tracking tag | MK geolocators, Lotek Wireless Inc., https://www.lotek.com/ | $ | Records and archives positional data on-board the tag. Typically requires recovery of the tag to acquire data. Can be paired with a variety of sensors (e.g. accelerometers). | Fort *et al.* (2014); Renedo *et al.* (2020) |
| *Radio frequency identification (RFID)* |  |  |  | Mainly used for long-term identification of a large number of individuals. Extremely short range of detection between RFID tags and readers. | Bonter & Bridge (2011); Kissling *et al.* (2014); Colin *et al.* (2019, 2021) |
|  | RFID tag | mic3® RFID transponder, Microsensys GmbH,  https://www.microsensys.de/en/ | Enquire for price | Passive unpowered tag that emits an electromagnetic signal for identification when powered by a reader in close proximity. Tags can be of extremely small sizes due to reliance on passive unpowered technology. |  |
|  | RFID reader | iID® PENmini or PENsolid readers, Microsensys GmbH,  https://www.microsensys.de/en/ | Enquire for price | For reading and writing of RFID tags. Tagged animals typically have to be in extremely close proximity to readers to be detected, due to short effective signal ranges. |  |
| **Animal monitoring** |  |  |  |  |  |
| *Camera trapping* |  |  |  | Cameras with motion sensors typically used to capture images or videos of animals in the wild to quantify a variety of behaviours such as foraging, sociality, periods of activity, or to take inventories of species abundance. | Caravaggi *et al.* (2017); Glover-Kapfer *et al.* (2019) |
|  | Trail camera | HP2W Hyperfire 2 professional white flash camera, Reconyx,  https://www.reconyx.com/ | $ | Motion sensor camera with a 0.2 second trigger speed, recommended for extreme environments. LED illumination with a white flash colour reaches out to 30 m or 100 feet at night. 1080P or 3MP image resolution, 720P HD video with audio and operating temperatures of −40 to +60 °C. |  |
|  | Trail camera | CORE No Glow trail camera, Bushnell, https://www.bushnell.com/ | $ | Motion sensor camera with no light emitted while capturing images or video. 80 feet night range, 24 MP image resolution and 1080P video resolution. |  |
| *Drone imaging* |  |  |  | Remotely piloted aircraft (drones) with mounted cameras typically used to take aerial images or videos of wildlife to gather wildlife population data. | Hodgson *et al.* (2018); Lyons *et al.* (2019) |
|  | Quadcopter with mounted camera | Phantom 4 Pro V2.0, DJI,  https://www.dji.com/ | $$$ | Remotely piloted aircraft with a mounted camera. Able to shoot 4K/60fps resolution videos and 20MP photos, with inbuilt OcuSync 2.0 video transmission technology that allows for 1080P livestreaming at a distance of up to 10 km. |  |
|  | Quadcopter with mounted camera | EVO II, Autel Robotics, https://auteldrones.com/ | $$$ | Remotely piloted aircraft with a mounted camera. Able to record 8K resolution videos and 48MP photos. |  |
| *Passive acoustic monitoring* |  |  |  | Used to measure, monitor, and determine the source(s) of sounds in the environment to collect acoustic data on animals, such as species presence, population sizes, and biotic interactions. |  |
|  | Acoustic device | Audiomoth, Open Acoustic Devices,  https://www.openacousticdevices.info/ | $ | Low-cost, full-spectrum acoustic logging device. | Hill *et al.* (2018) |
|  | Acoustic device | Song meter SM4 acoustic recorder, Wildlife Acoustics, Inc.,  https://www.wildlifeacoustics.com/ | $$ | Industry standard wildlife audio recorder. | Gibb *et al.* (2019); Sugai *et al.* (2019) |
| *Light detection and ranging (LiDAR) imaging* |  |  |  | Uses laser scanning technology to create a 3D representation of the surveyed environment, typically providing a full 360-degree view around the sensor. | Dalgleish *et al.* (2017); Chen *et al.* (2019) |
|  | LiDAR sensor | Puck lidar sensor (VLP-16 series), Velodyne Lidar, Inc.,  https://velodynelidar.com/ | $$$ | Compact and power-efficient, with a 100 m range. Versatile and able to be used across a variety of applications. |  |
|  | LiDAR sensor | OS2, Ouster, Inc., https://ouster.com/ | $$$$$ | High-resolution 3D sensing with a 200+ m range. Water-proof, robust to shock and vibration, and temperature-rated from −20 to +50 °C. |  |

§ Hardware prices: $ (<US$500), $$ (<US$1000), $$$ (<US$5000), $$$$ (<US$10,000), and $$$$$ (>US$10,000). Pricing is approximate and based on currency conversion as of 10-01-2022. Prices may also vary by region depending on shipping, clearance, and tax. Cost is provided for one unit (e.g. one tracking tag, one acoustic device), of which multiple may be required.

**Table S5.** (a) A brief overview of resources available for implementing mixed modelling approaches in behavioural ecotoxicology, and (b) examples of specific research questions and resources for testing potential effects of contaminant exposure on behavioural variation at the individual level.

| (a)  **Topic** | **Resources** | **R packages** |
| --- | --- | --- |
| Broad overviews of the utility of mixed models | Pinheiro & Bates (2000)  Bolker *et al.* (2009)  Zuur *et al.* (2009)  Kain *et al.* (2015)  Brooks *et al.* (2017)  Harrison *et al.* (2018) | *glmmTMB* ­– Magnusson *et al.* (2017)  *lmerTest –* Kuznetsova *et al.* (2013)  *lme4* – Bates *et al.* (2015)  *nlme* – Pinheiro *et al.* (2017)  See also: ‘GLMM FAQ’ –  https://bbolker.github.io/mixedmodels-misc/glmmFAQ.html |
| Good practice, avoiding pitfalls, and data visualisation | Zuur *et al.* (2010)  Kass *et al.* (2016)  Harrison *et al.* (2018)  Silk *et al.* (2020)  Westneat *et al.* (2020) | *ggplot2 –* Wickham & Chang (2015)  *performance –* Lüdecke *et al.* (2021) |
| Variance decomposition | Nakagawa & Schielzeth (2010)  Wilson *et al.* (2010)  Nakagawa & Schielzeth (2013)  Cleasby *et al.* (2015)  Allegue *et al.* (2017)  Houslay & Wilson (2017)  Nakagawa *et al.* (2017*a*)  O’Dea *et al.* (2021) | *rptR* – Stoffel *et al.* (2017)  *SQuiD –* Allegue *et al.* (2017) |
| Multimodal inference | Burnham *et al.* (2011)  Grueber *et al.* (2011)  Symonds & Moussalli (2011) | *MuMIn* – Bartoń (2020) |
| Bayesian approaches | Hadfield (2010)  Bürkner (2017, 2018)  Banner *et al.* (2020) | *brms –* Bürkner (2017, 2018)  *MCMCglmm –* Hadfield (2021) |
| (b)  **Research question** | **Definitions of the (co)variance term of interest** | **Resources** |
| Does responsiveness to environmental variation differ at the individual level between exposed and unexposed populations? | **Behavioural plasticity:** rapid changes in the behaviour of an individual in response to changing environmental conditions—e.g. exposed and unexposed treatment groups confronted with biotic (e.g. predation risk) and/or abiotic (e.g. temperature) stressors. Measured as the difference in the magnitude of behavioural adjustments (i.e. slopes) of individuals from different treatment groups. | Dingemanse *et al.* (2010)  Westneat *et al.* (2015) |
| Does exposure to environmental pollutants change how much individuals differ in their average behavioural response to a given context (e.g. response to a predator)? | **Behavioural types (personality/temperament):** among-individual differences in behaviour (e.g. activity) that are consistent over time and/or across contexts. Measured as the intercept of the individual’s behavioural reaction norm, and its respective position on the behavioural continuum (e.g. low–high activity continuum). | Réale *et al.* (2007)  Nakagawa *et al.* (2017*a*) |
| Does an individual’s behaviour become less or more predictable after exposure to an environmental pollutant? | **Behavioural predictability:** residual (i.e. within-individual) behavioural variance after accounting for the variation between individuals (i.e. behavioural type). | Cleasby *et al.* (2015) |
| Do individual differences in a specific behaviour predict differences in another behaviour tested under diverse environmental conditions? | **Behavioural syndromes:** correlation between two individual’s average behaviours (e.g. activity, risk-taking), with both behaviours being significantly repeatable after repeated measures. | Dingemanse & Dochterman (2013) |
| Do behavioural differences among individuals relate to differences in their physiological and life-history traits under diverse environmental conditions? | **Pace of Life Syndrome (POLS) hypothesis:** correlation between an individual’s average behaviour (e.g. activity) and averages of its other phenotypic traits (e.g. metabolic rate, growth rate), with all traits significantly repeatable after repeated measures. | Réale *et al.* (2010)  Dammhahn *et al.* (2018) |

**References**

Allegue, H., Araya-Ajoy, Y. G., Dingemanse, N. J., Dochtermann, N. A., Garamszegi, L. Z., Nakagawa, S., Réale, D., Schielzeth, H. & Westneat, D. F. (2017). Statistical Quantification of Individual Differences (SQuID): an educational and statistical tool for understanding multilevel phenotypic data in linear mixed models. *Methods in Ecology and Evolution* **8**, 257–267.

Asadpour, M., Tâche, F., Caprari, G., Karlen, W. & Siegwart, R. (2006). Robot-animal interaction: perception and behavior of InsBot. *International Journal of Advanced Robotic Systems* **3**, 93­–98.

Baktoft, H., Gjelland, K. Ø., Økland, F. & Thygesen, U. H. (2017). Positioning of aquatic animals based on time-of-arrival and random walk models using YAPS (Yet Another Positioning Solver). *Scientific Reports* **7**, 1–10.

Banner, K. M., Irvine, K. M. & Rodhouse, T. (2020). The use of Bayesian priors in Ecology: the good, the bad and the not great. *Methods in Ecology and Evolution* **11**, 882–889.

Bartoń, K. (2020). MuMIn: multi-model inference. R package version 1.43.17.

Bates, D., Machler, M., Bolker, B. M. & Walker, S. C. (2015). Fitting Linear Mixed-Effects Models using lme4. *Journal of Statistical Software* **67**, 1–48.

BehaviorCloud (in press). BehaviorCloud User Guide. See https://behaviorcloud.com/guide.html (accessed on 10 July 2021).

Beyan, C. & Browman, H. I. (2020). Setting the stage for the machine intelligence era in marine science. *ICES Journal of Marine Science* **77**, 1267–1273.

Blumstein, D. T. & Daniel, J. C. (2007). Quantifying behavior the JWatcher way. Sunderland, MA: Sinauer Associates Inc.

Bolger, D. T., Morrison, T. A., Vance, B., Lee, D. & Farid, H. (2012). A computer-assisted system for photographic mark–recapture analysis. *Methods in Ecology and Evolution* **3**, 813–822.

Bolker, B. M., Brooks, M. E., Clark, C. J., Geange, S. W., Poulsen, J. R., Stevens, M. H. H. & White, J. S. S. (2009). Generalized linear mixed models: a practical guide for ecology and evolution. *Trends in Ecology & Evolution* **24**, 127–135.

Bonter, D. N. & Bridge, E. S. (2011). Applications of radio frequency identification (RFID) in ornithological research: a review. *Journal of Field Ornithology* **82**, 1–10.

Branson, K., Robie, A. A., Bender, J., Perona, P. & Dickinson, M. H. (2009). High-throughput ethomics in large groups of *Drosophila*. *Nature Methods* **6**, 451–457.

Brooks, M. E., Kristensen, K., van Benthem, K. J., Magnusson, A., Berg, C. W., Nielsen, A., Skaug, H. J., Mächler, M. & Bolker, B. M. (2017). glmmTMB balances speed and flexibility among packages for zero-inflated generalized linear mixed modeling. *R Journal* **9**, 378–400.

Browning, E., Bolton, M., Owen, E., Shoji, A., Guilford, T. & Freeman, R. (2018). Predicting animal behaviour using deep learning: GPS data alone accurately predict diving in seabirds. *Methods in Ecology and Evolution* **9**, 681–692.

Bruneel, S., Verhelst, P., Reubens, J., Baetens, J. M., Coeck, J., Moens, T. & Goethals, P. (2020). Quantifying and reducing epistemic uncertainty of passive acoustic telemetry data from longitudinal aquatic systems. *Ecological Informatics* **59**, 101133.

Bürkner, P.-C. (2017). brms: an R package for Bayesian multilevel models using stan. *Journal of Statistical Software* **80**, 1–28.

Bürkner, P.-C. (2018). Advanced Bayesian Multilevel Modeling with the R Package brms. *R Journal* **10**, 395–411.

Burnham, K. P., Anderson, D. R. & Huyvaert, K. P. (2011). AIC model selection and multimodel inference in behavioral ecology: some background, observations, and comparisons. *Behavioral Ecology and Sociobiology* **65**, 23–35.

Campbell, H. A., Watts, M. E., Dwyer, R. G. & Franklin, C. E. (2012). V-Track: software for analysing and visualising animal movement from acoustic telemetry detections. *Marine and Freshwater Research* **63**, 815–820.

Caravaggi, A., Banks, P. B., Burton, A. C., Finlay, C. M., Haswell, P. M., Hayward, M. W., Rowcliffe, M. J. & Wood, M. D. (2017). A review of camera trapping for conservation behaviour research. *Remote Sensing in Ecology and Conservation* **3**, 109–122.

Cartlidge, R., Nugegoda, D. & Wlodkowic, D. (2015). GammarusChip: innovative lab-on-a-chip technology for ecotoxicological testing using the marine amphipod *Allorchestes compressa*. *Proceedings of SPIE, Bio-MEMS and Medical Microdevices II* **9518**, 951812.

Chen, J., Xu, H., Wu, J., Yue, R., Yuan, C. & Wang, L. (2019). Deer crossing road detection with roadside LiDAR sensor. *IEEE Access* **7**, 65944–65954.

Cheng, Y., Fiedler, W., Wikelski, M. & Flack, A. (2019). “Closer-to-home” strategy benefits juvenile survival in a long-distance migratory bird. *Ecology and Evolution* **9**, 8945–8952.

Christin, S., Hervet, É. & Lecomte, N. (2019). Applications for deep learning in ecology. *Methods in Ecology and Evolution* **10**, 1632–1644.

Cleasby, I. R., Nakagawa, S., Schielzeth, H. & Hadfield, J. (2015). Quantifying the predictability of behaviour: statistical approaches for the study of between‐individual variation in the within‐individual variance. *Methods in Ecology and Evolution* **6**, 27–37.

Colin, T., Meikle, W. G., Wu, X. & Barron, A. B. (2019). Traces of a neonicotinoid induce precocious foraging and reduce foraging performance in honey bees. *Environmental Science & Technology* **53**, 8252–8261.

Colin, T., Forster, C. C., Westacott, J., Wu, X., Meikle, W. G. & Barron, A. B. (2021). Effects of late miticide treatments on foraging and colony productivity of European honey bees (*Apis mellifera*). *Apidologie* **52**, 1­–19.

Colot, A., Caprari, G. & Siegwart, R. (2004). InsBot: design of an autonomous mini mobile robot able to interact with cockroaches. *IEEE International Conference on Robotics and Automation*, 2418–2423.

Cooke, S. J., Midwood, J. D., Thiem, J. D., Klimley, P., Lucas, M. C., Thorstad, E. B., Eiler, J., Holbrook, C. & Ebner, B. C. (2013). Tracking animals in freshwater with electronic tags: past, present and future. *Animal Biotelemetry* **1**, 5.

Crall, J. D., Gravish, N., Mountcastle, A. M. & Combes, S. A. (2015). BEEtag: a low-cost, image-based tracking system for the study of animal behavior and locomotion. *PLOS One* **10**, e0136487.

Crispim Junior, C. F., Pederiva, C. N., Bose, R. C., Garcia, V. A., Lino-de-Oliveira, C. & Marino-Neto, J. (2012). ETHOWATCHER: validation of a tool for behavioral and video-tracking analysis in laboratory animals. *Computers in Biology and Medicine* **42**, 257–264.

Dalgleish, F., Ouyang, B., Vuorenkoski, A., Ramos, B., Alsenas, G., Metzger, B., Cao, Z. & Principe, J. (2017). Undersea lidar imager for unobtrusive and eye safe marine wildlife detection and classification. *OCEANS 2017-Aberdeen* 1–5.

Dammhahn, M., Dingemanse, N. J., Niemelä, P. T. & Réale, D. (2018). Pace-of-life syndromes: a framework for the adaptive integration of behaviour, physiology and life history. *Behavioral Ecology and Sociobiology* **72**, 62–70.

Dingemanse, N. J., Kazem, A. J. N., Réale, D. & Wright, J. (2010). Behavioural reaction norms: animal personality meets individual plasticity. *Trends in Ecology & Evolution* **25**, 81–89.

Dingemanse, N. J. & Dochtermann, N. A. (2013). Quantifying individual variation in behaviour: mixed-effect modelling approaches. *Journal of Animal Ecology* **82**, 39–54.

Ditria, E. M., Lopez-Marcano, S., Sievers, M., Jinks, E. L., Brown, C. J. & Connolly, R. M. (2020). Automating the analysis of fish abundance using object detection: optimizing animal ecology with deep learning. *Frontiers in Marine Science* **7**, 429.

Donaldson, M. R., Hinch, S. G., Suski, C. D., Fisk, A. T., Heupel, M. R. & Cooke, S. J. (2014). Making connections in aquatic ecosystems with acoustic telemetry monitoring. *Frontiers in Ecology and the Environment* **12**, 565–573.

Dujon, A. M., Lindstrom, R. T. & Hays, G. C. (2014). The accuracy of Fastloc-GPS locations and implications for animal tracking. *Methods in Ecology and Evolution* **5**, 1162–1169.

Dunn, T. W., Marshall, J. D., Severson, K. S., Aldarondo, D. E., Hildebrand, D. G. C., Chettih, S. N., Wang, W. L., Gellis, A. J., Carlson, D. E., Aronov, D., Freiwald, W. A., Wang, F. & Ölveczky, B. P. (2021). Geometric deep learning enables 3D kinematic profiling across species and environments. *Nature Methods* **18**, 564–573.

Fort, J., Robertson, G. J., Grémillet, D., Traisnel, G. & Bustamante, P. (2014). Spatial ecotoxicology: migratory Arctic seabirds are exposed to mercury contamination while overwintering in the Northwest Atlantic. *Environmental Science & Technology* **48**, 11560–11567.

Francisco, F. A., Nührenberg, P. & Jordan, A. L. (2020). High-resolution, non-invasive animal tracking and reconstruction of local environment in aquatic ecosystems. *Movement Ecology* **8**, 1–12.

Friard, O. & Gamba, M. (2016). BORIS: a free, versatile open-source event-logging software for video/audio coding and live observations. *Methods in Ecology and Evolution* **7**, 1325–1330.

Gallois, B. & Candelier, R. (2021). FastTrack: an open-source software for tracking varying numbers of deformable objects. *PLOS Computational Biology* **17**, e1008697.

Gibb, R., Browning, E., Glover-Kapfer, P. & Jones, K. E. (2019). Emerging opportunities and challenges for passive acoustics in ecological assessment and monitoring. *Methods in Ecology and Evolution* **10**, 169–185.

Glover-Kapfer, P., Soto-Navarro, C. A. & Wearn, O. R. (2019). Camera-trapping version 3.0: current constraints and future priorities for development. *Remote Sensing in Ecology and Conservation* **5**, 209–223.

Graving, J. M., Chae, D., Naik, H., Li, L., Koger, B., Costelloe, B. R. & Couzin, I. D. (2019). DeepPoseKit, a software toolkit for fast and robust animal pose estimation using deep learning. *Elife* **8**, e47994.

Griffin, L. P., Smith, B. J., Cherkiss, M. S., Crowder, A. G., Pollock, C. G., Hillis-Starr, Z., Danylchuk, A. J. & Hart, K. M. (2020). Space use and relative habitat selection for immature green turtles within a Caribbean marine protected area. *Animal Biotelemetry* **8**, 1–13.

Grueber, C. E., Nakagawa, S., Laws, R. J. & Jamieson, I. G. (2011). Multimodel inference in ecology and evolution: challenges and solutions. *Journal of Evolutionary Biology* **24**, 699–711.

Hadfield, J. D. (2010). MCMC methods for multi-response generalized linear mixed models: the MCMCglmm R package. *Journal of Statistical Software* **33**, 1–22.

Hadfield, J. D. (2021). Package ‘MCMCglmm’. R package version 2.32.

Halloy, J., Sempo, G., Caprari, G., Rivault, C., Asadpour, M., Tâche, F., Said, I., Durier, V., Canonge, S., Amé, J. M., Detrain, C., Correll, N., Martinoli, A., Mondada, F., Siegwart, R. & Deneubourg, J. L. (2007). Social integration of robots into groups of cockroaches to control self-organized choices. *Science* **318**, 1155–1158.

Harrison, X. A., Donaldson, L., Correa-Cano, M. E., Evans, J., Fisher, D. N., Goodwin, C. E., Robinson, B. S., Hodgson, D. J. & Inger, R. (2018). A brief introduction to mixed effects modelling and multi-model inference in ecology. *PeerJ* **6**, e4794.

Haulsee, D. E., Fox, D. A., Breece, M. W., Clauss, T. M. & Oliver, M. J. (2016). Implantation and recovery of long-term archival transceivers in a migratory shark with high site fidelity. *PLOS One* **11**, e0148617.

Hays, G. C., Ferreira, L. C., Sequeira, A. M., Meekan, M. G., Duarte, C. M., Bailey, H., Bailleul, F., Bowen, W. D., Caley, M. J., Costa, D. P. & Eguíluz, V. M. (2016). Key questions in marine megafauna movement ecology. *Trends in Ecology & Evolution* **31**, 463–475.

Hebert, L., Ahamed, T., Costa, A. C., O’Shaughnessy, L. & Stephens, G. J. (2021). WormPose: image synthesis and convolutional networks for pose estimation in *C*. *elegans*. *PLOS Computational Biology* **17**, e1008914.

Hill, A. P., Prince, P., Piña Covarrubias, E., Doncaster, C. P., Snaddon, J. L. & Rogers, A. (2018). AudioMoth: evaluation of a smart open acoustic device for monitoring biodiversity and the environment. *Methods in Ecology and Evolution* **9**, 1199–1211.

Hodgson, J. C., Mott, R., Baylis, S. M., Pham, T. T., Wotherspoon, S., Kilpatrick, A. D., Raja Segaran, R., Reid, I., Terauds, A. & Koh, L. P. (2018). Drones count wildlife more accurately and precisely than humans. *Methods in Ecology and Evolution* **9**, 1160–1167.

Houslay, T. & Wilson, A. (2017). Avoiding the misuse of BLUP in behavioral ecology. *Behavioral Ecology* **28**, 948–952.

Hussey, N. E., Kessel, S. T., Aarestrup, K., Cooke, S. J., Cowley, P. D., Fisk, A. T., Harcourt, R. G., Holland, K. N., Iverson, S. J., Kocik, J. F. & Flemming, J. E. M. (2015). Aquatic animal telemetry: a panoramic window into the underwater world. *Science* **348**, 1255642.

Jackson, B. E., Evangelista, D. J., Ray, D. D. & Hedrick, T. L. (2016). 3D for the people: multi-camera motion capture in the field with consumer-grade cameras and open source software. *Biology Open* **5**, 1334–1342.

Javer, A., Currie, M., Lee, C. W., Hokanson, J., Li, K., Martineau, C. N., Yemini, E., Grundy, L. J., Li, C., Ch’ng, Q., Schafer, W. R., Nollen, E. A. A., Kerr, R. & Brown, A. E. X. (2018). An open-source platform for analyzing and sharing worm-behavior data. *Nature Methods* **15**, 645–646.

Jolles, J. W. (2020). Pirecorder: controlled and automated image and video recording with the Raspberry Pi. *Journal of Open Source Software* **5**, 2584.

Jolles, J. W. (2021). Broad-scale applications of the Raspberry Pi: a review and guide for biologists. *Methods in Ecology and Evolution* **12**, 1562–1579.

Joo, R., Boone, M. E., Clay, T. A., Patrick, S. C., Clusella-Trullas, S. & Basille, M. (2020). Navigating through the R packages for movement. *Journal of Animal Ecology* **89**, 248–267.

Kain, M. P., Bolker, B. M. & McCoy, M. (2015). A practical guide and power analysis for GLMMs: detecting among treatment variation in random effects. *PeerJ* **3**, e1226.

Kass, R. E., Caffo, B. S., Davidian, M., Meng, X. L., Yu, B. & Reid, N. (2016). Ten simple rules for effective statistical practice. *PLOS Computational Biology* **12**, e1004961.

Kays, R., Crofoot, M. C., Jetz, W. & Wikelski, M. (2015). Terrestrial animal tracking as an eye on life and planet. *Science* **348**, aaa2478.

Kissling, W. D., Pattemore, D. E. & Hagen, M. (2014). Challenges and prospects in the telemetry of insects. *Biological Reviews* **89**, 511–530.

Kopman, V. & Porfiri, M. (2013). Design, modeling, and characterization of a miniature robotic fish for research and education in biomimetics and bioinspiration. *IEEE/ASME Transactions on Mechatronics* **18**, 471–483.

Kopman, V., Laut, J., Polverino, G. & Porfiri, M. (2013). Closed-loop control of zebrafish response using a bioinspired robotic-fish in a preference test. *Journal of the Royal Society Interface* **10**, 20120540.

Kuznetsova, A., Brockhoff, P. B. & Christensen, R. H. B. (2013). lmerTest: tests for random and fixed effects for linear mixed effect models. R package version 2–6.

Lennox, R. J., Aarestrup, K., Cooke, S. J., Cowley, P. D., Deng, Z. D., Fisk, A. T., Harcourt, R. G., Heupel, M., Hinch, S. G., Holland, K. N. & Hussey, N. E. (2017). Envisioning the future of aquatic animal tracking: technology, science, and application. *BioScience* **67**, 884–896.

Lin, Y., Hsiung, J., Piersall, R., White, C., Lowe, C. G. & Clark, C. M. (2017). A multi-autonomous underwater vehicle system for autonomous tracking of marine life. *Journal of Field Robotics* **34**, 757–774.

Loligo-Systems (in press). Quick guide for LoliTrack 5, 1–4.

Lüdecke, D., Ben-Shachar, M. S., Patil, I., Waggoner, P. & Makowski, D. (2021). performance: an R package for assessment, comparison and testing of statistical models. *Journal of Open Source Software* **6**, 3139.

Lyons, M. B., Brandis, K. J., Murray, N. J., Wilshire, J. H., McCann, J. A., Kingsford, R. T. & Callaghan, C. T. (2019). Monitoring large and complex wildlife aggregations with drones. *Methods in Ecology and Evolution* **10**, 1024–1035.

Magnusson, A., Skaug, H., Nielsen, A., Berg, C., Kristensen, K., Maechler, M., Van Bentham, K., Bolker, B., Brooks, M. & Brooks, M. M. (2017). Package ‘glmmTMB’. R package version 0.2.0.

Meijering, E., Dzyubachyk, O. & Smal, I. (2012). Methods for cell and particle tracking. *Methods in Enzymology* **504**, 183–200.

Nakagawa, S. & Schielzeth, H. (2010). Repeatability for Gaussian and non-Gaussian data: a practical guide for biologists. *Biological Reviews* **85**, 935–956.

Nakagawa, S. & Schielzeth, H. (2013). A general and simple method for obtaining *R*^2^ from generalized linear mixed-effects models. *Methods in Ecology and Evolution* **4**, 133–142.

Nakagawa, S., Johnson, P. C. D. & Schielzeth, H. (2017*a*). The coefficient of determination *R*^2^ and intraclass correlation coefficient from generalized linear mixed-effects models revisited and expanded. *Journal of the Royal Society Interface* **14**, 20170213.

Nath, T., Mathis, A., Chen, A. C., Patel, A., Bethge, M. & Mathis, M. W. (2019). Using DeepLabCut for 3D markerless pose estimation across species and behaviors. *Nature Protocols* **14**, 2152–2176.

Nathan, R., Monk, C., Arlinghaus, R., Adam, T., Alós, J., Assaf, M., Baktoft, H., Beardsworth, C. E., Bertram, M. G., Bijleveld, A., Brodin, T., Brooks, J., Campos-Candela, A., Cooke, S. J., Gjelland, K. J., *et al*. (2022). Big-data approaches lead to an increased understanding of the ecology of animal movement. *Science* **375**, eabg1780.

Norouzzadeh, M. S., Nguyen, A., Kosmala, M., Swanson, A., Palmer, M. S., Packer, C. & Clune, J. (2018). Automatically identifying, counting, and describing wild animals in camera-trap images with deep learning. *Proceedings of the National Academy of Sciences of the United States of America* **115**, E5716–E5725.

Norouzzadeh, M. S., Morris, D., Beery, S., Joshi, N., Jojic, N. & Clune, J. (2021). A deep active learning system for species identification and counting in camera trap images. *Methods in Ecology and Evolution* **12**, 150–161.

Nussbaum-Krammer, C. I., Neto, M. F., Brielmann, R. M., Pedersen, J. S. & Morimoto, R. I. (2015). Investigating the spreading and toxicity of prion-like proteins using the metazoan model organism *C. elegans*. *Journal of Visualized Experiments* **95**, e52321.

O’Dea, R. E., Noble, D. W. A. & Nakagawa, S. (2022). Unifying individual differences in personality, predictability and plasticity: a practical guide. *Methods in Ecology and Evolution* **13**, 278–293.

Pérez-Escudero, A., Vicente-Page, J., Hinz, R. C., Arganda, S. & de Polavieja, G. G. (2014). idTracker: tracking individuals in a group by automatic identification of unmarked animals. *Nature Methods* **11**, 743–748.

Pereira, T. D., Aldarondo, D. E., Willmore, L., Kislin, M., Wang, S. S. H., Murthy, M. & Shaevitz, J. W. (2019). Fast animal pose estimation using deep neural networks. *Nature Methods* **16**, 117–125.

Pereira, T. D., Tabris, N., Li, J., Ravindranath, S., Papadoyannis, E. S., Wang, Z. Y., Turner, D. M., McKenzie-Smith, G., Kocher, S. D., Falkner, A. L., Shaevitz, J. W. & Murthy, M. (2020). SLEAP: multi-animal pose tracking. *bioRxiv*.

Pinheiro, J. C. & Bates, D. M. (2000). Linear mixed-effects models: basic concepts and examples. In: Mixed-effects models in S and S-PLUS. New York, NY: Springer.

Pinheiro, J., Bates, D., DebRoy, S., Sarkar, D. & R Core Team. (2017). nlme: Linear and nonlinear mixed effects models. R Package version 3.1-131.

Polverino, G., Karakaya, M., Spinello, C., Soman, V. R. & Porfiri, M. (2019). Behavioural and life-history responses of mosquitofish to biologically inspired and interactive robotic predators. *Journal of the Royal Society Interface* **16**, 20190359.

Polverino, G., Soman, V. R., Karakaya, M., Gasparini, C., Evans, J. P. & Porfiri, M. (2022). Ecology of fear in highly invasive fish revealed by robots. *iScience* **25**, 103529.

Réale, D., Reader, S. M., Sol, D., McDougall, P. T. & Dingemanse, N. J. (2007). Integrating animal temperament within ecology and evolution. *Biological Reviews* **82**, 291–318.

Réale, D., Garant, D., Humphries, M. M., Bergeron, P., Careau, V. & Montiglio, P. O. (2010). Personality and the emergence of the pace-of-life syndrome concept at the population level. *Philosophical Transactions of the Royal Society B: Biological Sciences* **365**, 4051–4063.

Renedo, M., Amouroux, D., Albert, C., Bérail, S., Bråthen, V. S., Gavrilo, M., Grémillet, D., Helgason, H. H., Jakubas, D., Mosbech, A., Strøm, H., Tessier, E., Wojczulanis-Jakubas, K., Bustamante, P., *et al*. (2020). Contrasting spatial and seasonal trends of methylmercury exposure pathways of Arctic seabirds: combination of large-scale tracking and stable isotopic approaches. *Environmental Science & Technology* **54**, 13619­–13629.

Rodriguez, A., Zhang, H., Klaminder, J., Brodin, T., Andersson, P. L. & Andersson, M. (2018). *ToxTrac*: a fast and robust software for tracking organisms. *Methods in Ecology and Evolution* **9**, 460–464.

Romero-Ferrero, F., Bergomi, M. G., Hinz, R. C., Heras, F. J. H. & de Polavieja, G. G. (2019). idtracker.ai: tracking all individuals in small or large collectives of unmarked animals. *Nature Methods* **16**, 179–182.

Schneider, C. A., Rasband, W. S. & Eliceiri, K. W. (2012). NIH Image to ImageJ: 25 years of image analysis. *Nature Methods* **9**, 671–675.

Sergio, F., Tanferna, A., Blas, J., Blanco, G. & Hiraldo, F. (2019). Reliable methods for identifying animal deaths in GPS-and satellite-tracking data: review, testing, and calibration. *Journal of Applied Ecology* **56**, 562–572.

Silk, M. J., Harrison, X. A. & Hodgson, D. J. (2020). Perils and pitfalls of mixed-effects regression models in biology. *PeerJ* **8**, e9522.

Smith, M. L., Davidson, J. D., Wild, B., Dormagen, D. M., Landgraf, T. & Couzin, I. D. (2021). The dominant axes of lifetime behavioral variation in honey bees. *bioRxiv.*

Spinello, C., Macrì, S. & Porfiri, M. (2013). Acute ethanol administration affects zebrafish preference for a biologically inspired robot. *Alcohol* **47**, 391–398.

Spink, A. J., Tegelenbosch, R. A. J., Buma, M. O. S. & Noldus, L. P. J. J. (2001). The EthoVision video tracking system—a tool for behavioral phenotyping of transgenic mice. *Physiology & Behavior* **73**, 731–744.

Sridhar, V. H., Roche, D. G. & Gingins, S. (2019). Tracktor: image-based automated tracking of animal movement and behaviour. *Methods in Ecology and Evolution* **10**, 815–820.

Stoffel, M. A., Nakagawa, S. & Schielzeth, H. (2017). rptR: repeatability estimation and variance decomposition by generalized linear mixed-effects models. *Methods in Ecology and Evolution* **8**, 1639–1644.

Stowers, J. R., Hofbauer, M., Bastien, R., Griessner, J., Higgins, P., Farooqui, S., Fischer, R. M., Nowikovsky, K., Haubensak, W., Couzin, I. D., Tessmar-Raible, K. & Straw, A. D. (2017). Virtual reality for freely moving animals. *Nature Methods* **14**, 995–1002.

Sugai, L. S. M., Silva, T. S. F., Ribeiro Jr, J. W. & Llusia, D. (2019). Terrestrial passive acoustic monitoring: review and perspectives. *BioScience* **69**, 15–25.

Symonds, M. R. E. & Moussalli, A. (2011). A brief guide to model selection, multimodel inference and model averaging in behavioural ecology using Akaike’s information criterion. *Behavioral Ecology and Sociobiology* **65**, 13–21.

Taylor, P., Crewe, T., Mackenzie, S., Lepage, D., Aubry, Y., Crysler, Z., Finney, G., Francis, C., Guglielmo, C., Hamilton, D. & Holberton, R. (2017). The Motus Wildlife Tracking System: a collaborative research network to enhance the understanding of wildlife movement. *Avian Conservation and Ecology* **12**, 8.

Thomson, J. A., Börger, L., Christianen, M. J. A., Esteban, N., Laloë, J. O. & Hays, G. C. (2017). Implications of location accuracy and data volume for home range estimation and fine-scale movement analysis: comparing Argos and Fastloc-GPS tracking data. *Marine Biology* **164**, 204.

Toledo, S., Kishon, O., Orchan, Y., Shohat, A. & Nathan, R. (2016). Lessons and experiences from the design, implementation, and deployment of a wildlife tracking system. *2016 IEEE International Conference on Software Science, Technology and Engineering (SWSTE)* 51–60.

Toledo, S., Shohami, D., Schiffner, I., Lourie, E., Orchan, Y., Bartan, Y. & Nathan, R. (2020). Cognitive map–based navigation in wild bats revealed by a new high-throughput tracking system. *Science* **369**, 188–193.

Walter, T. & Couzin, I. D. (2021). TRex, a fast multi-animal tracking system with markerless identification, and 2D estimation of posture and visual fields. *eLife* **10**, e64000.

Wario, F., Wild, B., Couvillon, M. J., Rojas, R. & Landgraf, T. (2015). Automatic methods for long-term tracking and the detection and decoding of communication dances in honeybees. *Frontiers in Ecology and Evolution* **3**, 103.

Wark, J. D., Cronin, K. A., Niemann, T., Shender, M. A., Horrigan, A., Kao, A. & Ross, M. R. (2019). Monitoring the behavior and habitat use of animals to enhance welfare using the ZooMonitor app. *Animal Behavior and Cognition* **6**, 158–167.

Weiser, A. W., Orchan, Y., Nathan, R., Charter, M., Weiss, A. J. & Toledo, S. (2016). Characterizing the accuracy of a self-synchronized reverse-GPS wildlife localization system. *2016 15th ACM/IEEE International Conference on Information Processing in Sensor Networks (IPSN)* 1–12.

Westneat, D. F., Wright, J. & Dingemanse, N. J. (2015). The biology hidden inside residual within‐individual phenotypic variation. *Biological Reviews* **90**, 729–743.

Westneat, D. F., Araya-Ajoy, Y. G., Allegue, H., Class, B., Dingemanse, N., Dochtermann, N. A., Garamszegi, L. Z., Martin, J. G. A., Nakagawa, S., Réale, D. & Schielzeth, H. (2020). Collision between biological process and statistical analysis revealed by mean centring. *Journal of Animal Ecology* **89**, 2813–2824.

Wickham, H. & Chang, W. (2015). Package ‘ggplot2’. R package version 1.0.0.

Wild, B., Dormagen, D. M., Zachariae, A., Smith, M. L., Traynor, K. S., Brockmann, D., Couzin, I. D. & Landgraf, T. (2021). Social networks predict the life and death of honey bees. *Nature Communications* **12**, 1110.

Wilson, A. J., Réale, D., Clements, M. N., Morrissey, M. M., Postma, E., Walling, C. A., Kruuk, L. E. B. & Nussey, D. H. (2010). An ecologist’s guide to the animal model. *Journal of Animal Ecology* **79**, 13–26.

Yamanaka, O. & Takeuchi, R. (2018). UMATracker: an intuitive image-based tracking platform. *Journal of Experimental Biology* **221**, jeb182469.

Zimmerman, P. H., Bolhuis, J. E., Willemsen, A., Meyer, E. S. & Noldus, L. P. J. J. (2009). The Observer XT: a tool for the integration and synchronization of multimodal signals. *Behavior Research Methods* **41**, 731–735.

Zuur, A., Ieno, E. N., Walker, N., Saveliev, A. A. & Smith, G. M. (2009). Mixed effects models and extensions in ecology with R. New York, NY: Springer.

Zuur, A. F., Ieno, E. N. & Elphick, C. S. (2010). A protocol for data exploration to avoid common statistical problems. *Methods in Ecology and Evolution* **1**, 3­–14.
